# Insights into the transcriptional regulation of *CD22* in B cell chronic lymphocytic leukemia

**DOI:** 10.1016/j.jbc.2025.108386

**Published:** 2025-03-05

**Authors:** Bayarmaa Enkhbayar, Shao-Chia Lu, Ho-Yang Tsai, Suh-Yuen Liang, Shang-Ju Wu, Kuo-I Lin, Takashi Angata

**Affiliations:** 1 Institute of Biological Chemistry, Academia Sinica, Taipei, Taiwan; 2 Institute of Biochemical Sciences, National Taiwan University, Taipei, Taiwan; 3 Chemical Biology and Molecular Biophysics Program, Taiwan International Graduate Program, Academia Sinica, Taipei, Taiwan; 4 Genomics Research Center, Academia Sinica, Taipei, Taiwan; 5 Division of Hematology, Department of Internal Medicine, National Taiwan University Hospital, Taipei, Taiwan

**Keywords:** CD22, chronic lymphocytic leukemia, IRF4, PU.1, Spi-B, transcriptional regulation

## Abstract

CD22 (also known as Siglec-2) is a member of the Siglec family of glycan-recognition proteins and functions as a negative regulator of the B-cell receptor-mediated calcium signaling. Although the low level of CD22 expression on the B cells in patients with chronic lymphocytic leukemia (CLL) has been documented, CD22’s role and its downregulation mechanism in CLL are yet to be fully studied. In this study, we confirmed that the surface CD22 protein and its mRNA are downregulated in the B cells of CLL patients. We analyzed a public transcriptomic dataset and found that the *CD22* mRNA level is negatively associated with the prognosis of patients with CLL. To investigate the mechanism of CD22 downregulation, we characterized the minimal promoter of the human *CD22* gene required for its transcriptional activation in B cell lines. We employed an unbiased proteomic approach to identify several transcription factors binding to the minimal *CD22* promoter, including PU.1, Spi-B, and IRF4. The chromatin immunoprecipitation-quantitative PCR revealed that PU.1 was enriched in a CD22-high cell line, while IRF4 was enriched in a CD22-low cell line. We then conducted overexpression/knockout/knockdown experiments, which validated that PU.1 and Spi-B positively, and IRF4 negatively, regulate *CD22* transcription. Our study thus provides insights into the transcriptional regulation of *CD22* and the mechanism by which *CD22* expression is downregulated in the B cells of patients with CLL.

Chronic lymphocytic leukemia (CLL) is the most common form of leukemia in adults (1, 2). While it is generally indolent, a subset of CLL patients (*e.g.*, those with unmutated immunoglobulin heavy chain variable region (*IGHV*)) has a poorer prognosis (3, 4). Since the malignant B cells in CLL require B cell receptor (BCR) signaling for survival (5), targeting the BCR signaling pathway using small-molecule inhibitors, such as Ibrutinib (targeting Bruton’s tyrosine kinase) and Idelalisib (targeting phosphatidylinositol 3-kinase δ), has proven successful in the management of CLL (6, 7). However, resistance to these inhibitors can develop during the treatment (8), and alternative therapeutic approaches are being explored (9, 10).

CD22, also known as Siglec-2, is a member of the Siglec family of glycan-recognition proteins expressed primarily on B lymphocytes and negatively modulates BCR signaling (11, 12). The cross-linking of CD22 can induce apoptosis through the downregulation of the anti-apoptotic member(s) (13) or the upregulation of a pro-apoptotic member (14) of the BCL2 family. Immunotherapies targeting CD22, including unmodified or drug-conjugated anti-CD22 antibodies and chimeric antigen receptors against CD22, have been developed as treatments against B cell leukemia and lymphoma (15, 16). CD22 has been found to be downregulated in B cells derived from CLL patients (17, 18, 19), but the clinical significance and the mechanism underlying this observation remain unclear. The mechanism of CD22 downregulation in CLL B cells is of clinical interest, as the CD22 protein’s low expression may be correlated with the inefficiency of CD22-targeting drugs against CLL (20, 21).

The CD22 protein expression can be regulated at multiple levels, including transcriptional, post-transcriptional, and posttranslational levels. For example, past research has found post-transcriptional regulation of CD22 by microRNAs (22) and posttranslational regulation of CD22 protein by internalization through the clathrin-mediated pathway (23). Nevertheless, our preliminary analysis of a published transcriptomic dataset (Gene Expression Omnibus accession number: GSE36907) indicated that the *CD22* mRNA level is lower in the B cells of CLL patients than in the CD5^+^ B cells of healthy donors (*i.e.*, the putative normal counterpart of CLL B cells (24)), suggesting that transcriptional regulation may play a major role in CD22 protein expression. Based on DNA sequences, a previous study offered a preliminary characterization of human *CD22* promoter and predictions for the transcription factor–binding sites (25). Another previous study reported bioinformatic analysis of putative mouse *Cd22* promoter region and predicted putative-binding sites for several transcription factors (26). However, it remains unknown which transcription factors are involved in the transactivation or repression of human *CD22* and whether any of these factors can explain the downregulation of CD22 protein in the B cells of CLL patients.

To understand the transcriptional regulation mechanism of human *CD22*, we defined the minimal promoter of human *CD22* and identified the transcription factors that bind to it using an unbiased proteomic approach. The cotransfection experiment and chromatin immunoprecipitation-quantitative PCR (ChIP-qPCR) analysis using B cell lines revealed PU.1 and Spi-B as positive regulators and IRF4 as a negative regulator of *CD22* transcriptional regulation in B cells. The genetic manipulation of these transcription factors in the B cell lines revealed their possible roles in the downregulation of *CD22* in CLL B cells. Our study thus illustrates the basic mechanism of *CD22* transcription in B cells and its dysregulation in CLL B cells.

## Results

### Downregulation of *CD22* at the transcriptional level in B cells from CLL patients correlates with poor prognosis

**Figure 1:**
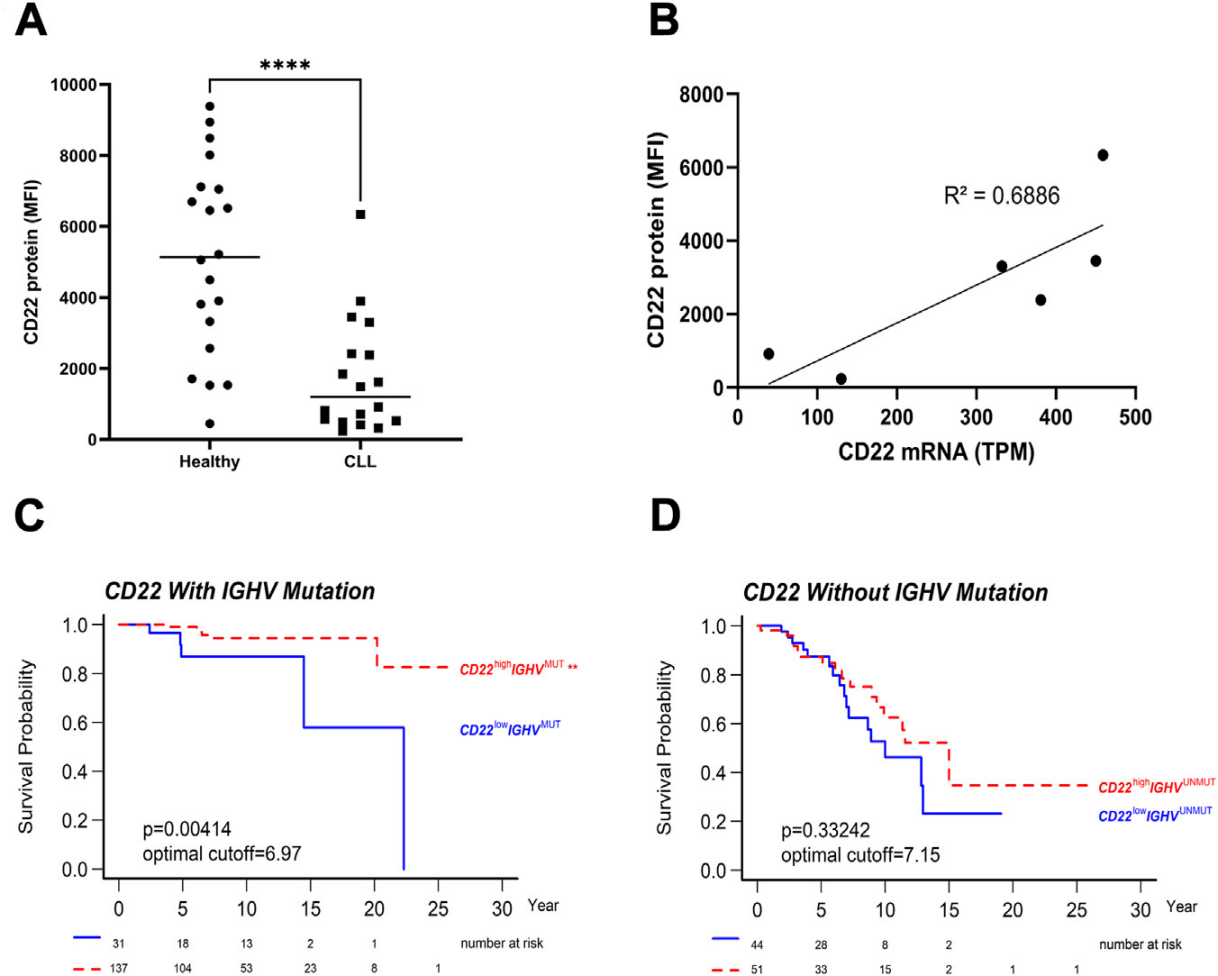
**Downregulation of CD22 in the B cells of CLL patients and its association with disease prognosis.***A*, expression of the CD22 protein on the B cells from patients with CLL and healthy donors. *n* = 20 (healthy donors) and 18 (CLL patients), respectively. ∗∗∗∗*p* < 0.0001 (Mann-Whitney test). *B*, correlation between the *CD22* transcript and the protein expression levels in Taiwanese patients with CLL. *C* and *D*, the transcriptomic data of Spanish CLL patients were stratified into (*C*) *IGHV*-mutated (*n* = 168) and (*D*) *IGHV*-unmutated (*n* = 95) datasets and analyzed for the correlation between the *CD22* transcript level and patient survival. The optimal cut-off was determined for each group with the cutp function in survMisc (60), an R package. MFI, median fluorescence intensity; TPM, transcripts per kilobase million.

To confirm the previous reports that CD22 is downregulated in the B cells of CLL patients, we used flow cytometry to analyze the peripheral blood B cells from CLL patients and those from healthy donors. Indeed, we found that the CD22 protein expression level on the B cells of CLL patients was significantly lower than that on the B cells from healthy donors (Fig. 1*A*; *p* < 0.0001, Mann-Whitney test). Moreover, we observed a positive correlation (R^2^ = 0.69; R = 0.83) between the *CD22* transcript (by RNA-Seq) and the CD22 protein levels (by flow cytometry) for the patients with CLL (Fig. 1*B*), suggesting that the downregulation of CD22 expression in CLL B cells is likely regulated at the transactional level.

Next, to check whether the *CD22* expression level is associated with the prognosis of CLL patients, we employed a publicly available dataset for Spanish CLL patients (*n* = 263) (27, 28). We stratified the patients in this dataset into *IGHV*-mutated (mCLL, *n* = 168) and *IGHV*-unmutated (uCLL, *n* = 95), as the prognoses for these groups are different (3, 4) and could confound the association analysis. Among the patients with mCLL, the prognosis of the *CD22*^low^ patients was significantly poorer than that of the *CD22*^high^ patients (Fig. 1*C*; *p* = 0.00414, log-rank test). Though a trend in the same direction was observed among the patients with uCLL, it did not attain statistical significance (Fig. 1*D*; *p* = 0.332, log-rank test). These observations are consistent with CD22 protein’s role in downregulating the BCR signaling, which is essential for the survival of CLL B cells (5).

### Characterization of minimal human *CD22* promoter sequence

**Figure 2:**
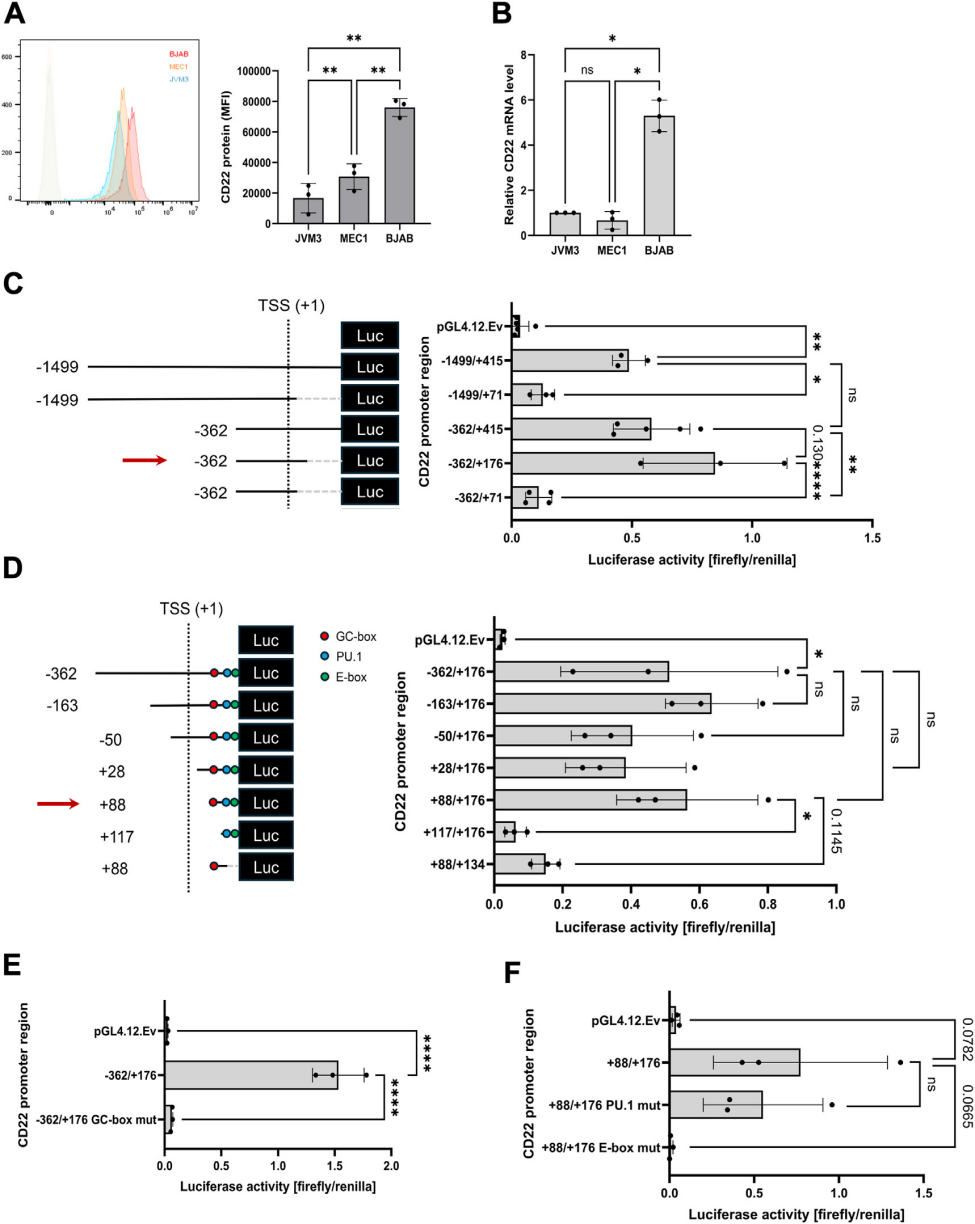
**Characterization of *CD22* minimal promoter.***A*, CD22 surface protein expression and (*B*) *CD22* mRNA levels in two CLL cell lines (JVM-3 and MEC-1) and one non-CLL cell line (BJAB). *C*, deletion of *CD22* promoter to identify the regions required for its transcriptional activation in BJAB cells. The firefly luciferase activities were normalized with a codelivered *Renilla* luciferase reporter under the PGK1 promoter. *D*, fine mapping of the region required for *CD22* transcriptional activation in BJAB cells. *E* and *F*, luciferase assay with (*E*) mutated GC-box and (*F*) mutated E-box or PU.1-binding site in the *CD22* promoter. ∗*p* < 0.05, ∗∗*p* < 0.01, ∗∗∗∗*p* < 0.0001, ns: *p* > 0.2; *p* value is indicated when 0.2 > *p* > 0.05 (ANOVA with Tukey’s *post hoc* test). *n* = 3 (independent experimental replicates).

The results suggest that *CD22* is downregulated in the B cells of CLL patients at the transcriptional level. However, the mechanism of the transcriptional regulation of *CD22* is poorly understood. To characterize and compare the *CD22* promoter activity in cell culture, we analyzed the CD22 expression in two CLL cell lines (JVM-3 and MEC-1) and a non-CLL cell line (BJAB, a human Burkitt lymphoma cell line) at both the protein and mRNA levels (Fig. 2, *A* and *B*). The two CLL cell lines, we found, have lower CD22 expressions than the control BJAB cell line, mirroring the trend of the CD22 expression observed in B cells from primary CLL patients and healthy donors.

A previous study characterized the *CD22* promoter region and identified its transcriptional start site (TSS; *i.e.*, the first nucleotide of exon 1) (25). However, the exon 1 sequence described in that study does not match the sequence in the reference human *CD22* cDNA (GenBank NM_001771) or other transcripts (Fig. S1). To characterize the *CD22* promoter, we prepared a reporter construct containing 1499 nucleotides upstream and 415 nucleotides downstream of the reported TSS (−1499/+415; corresponding to GRCh38/hg38 chr19:35,327,524-35,329,430) by performing PCR amplification of this region from the genomic DNA of JVM-3 cells (Fig. S2*A*). This segment, we found, exhibited transcriptional activity in the BJAB cell line that expresses CD22 at a high level (indicating active transcriptional machinery for CD22) (Fig. 2*C*).

To narrow down the core *CD22* promoter sequence for identifying the critical transcription factor–binding sites, we constructed several deletion variants of the *CD22* promoter in the reporter plasmid. In the first round of deletion, interestingly, we found that the *CD22* promoter region of the nucleotide sequence from −362 to +176 (corresponding to GRCh38/hg38 chr19:35,328,655-35,329,191) exhibited the highest reporter activity in the BJAB cells, while the *CD22* promoter variants with 3′ end deletion to +71 that was previously reported in (25) showed significantly lower reporter activity (Fig. 2*C*).

Starting from the −362/+176 region of the *CD22* promoter, we further deleted the promoter to identify the minimal promoter of *CD22* (Fig. 2*D*). We found that +88 to +176 of the *CD22* promoter (corresponding to GRCh38/hg38 chr19:35,329,103-35,329,191; with the 3′ overlapping with the 5′ end of most *CD22* transcripts) is required for the maximum transactivation of the reporter gene in the BJAB cells. By comparing the genomic DNA sequences (29), we found that there are GC-box, PU.1, and E-box elements that are conserved between different mammals (Fig. S3). Deleting these DNA elements in the *CD22* promoter also reduced promoter activity (Fig. 2*D*). Further, mutations in these elements attenuated the reporter gene expression (or showed a trend toward attenuation) in BJAB cells (Figs. 2, *E* and *F*; S2*B*), suggesting that these elements may be involved in the transactivation of *CD22*.

### Identification of the transcription factors that bind to the minimal *CD22* promoter using an unbiased proteomics approach

**Figure 3:**
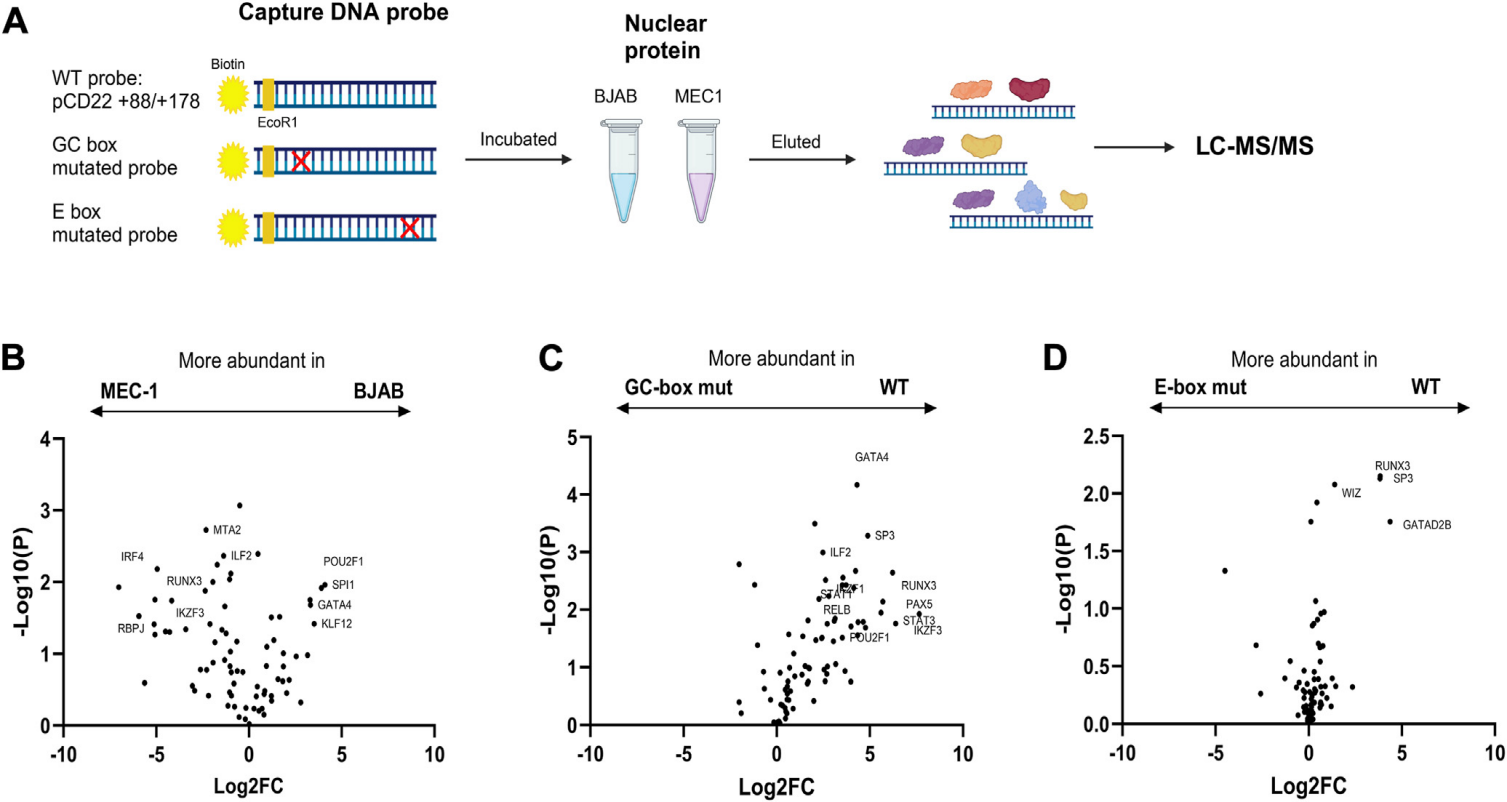
**Identification of the candidate transcription factors by DNA pulldown proteomics.***A*, biotinylated DNA fragment corresponding to the minimal *CD22* promoter (+88/+176) was immobilized on magnetic beads and used for the pulldown of proteins from the nuclear extracts of BJAB (CD22-high) and MEC-1 (CD22-low) cell lines. The DNA–protein complex was released by cleavage with EcoRI (extended at 5′ end), digested with LysC and trypsin, and the proteins therein were identified by liquid chromatography-coupled tandem mass spectrometry (LC-MS^2^). The amount of each protein (by LFQ using MaxQuant) was normalized by the total amount of proteins in the sample. The comparisons of proteins (*B*) extracted from BJAB *versus* MEC-1, (*C*) extracted from BJAB with WT *versus* GC-box mutant probes, and (*D*) extracted from BJAB with WT *versus* E-box mutant probes are shown. The proteins whose abundance in one sample exceeds twice that in the other sample and are statistically significant (nominal *p* < 0.05) are labeled.

We hypothesized that the B cells from CLL patients may lack a transcriptional activator that upregulates *CD22* transcription or overexpress a transcriptional repressor that downregulates it. To identify the transcriptional activators/repressors that bind to the minimal promoter of human *CD22* in an unbiased manner, we adapted a proteomic approach (30, 31) by using the *CD22* minimal promoter (+88/+178) as a bait to pull down the transcription factors from the nuclear extracts of the BJAB and MEC-1 cell lines (Fig. 3*A*). We also prepared two probes with mutations in the GC-box and E-box elements and used them in the pulldown experiments in parallel. The proteins pulled down with these DNA probes were identified by proteomic analysis with liquid chromatography-coupled tandem mass spectrometry and quantified using MaxQuant software (32). We annotated the identified proteins with PANTHER (33), filtered the proteins involved in transcriptional regulation (*i.e.*, PANTHER Protein Class annotation contains “transcription”), and compared their relative abundances [1] between the proteins pulled down from the BJAB and MEC-1 and [2] between the proteins pulled down with the WT and mutant probes (Figs. 3, *B*–*D*, and S4).

We found that the transcription factors, such as GATA4, POU2F1 (aka Oct-1), and SPI1 (aka PU.1), were enriched in the samples pulled down from BJAB, whereas RUNX3, IRF4, and RBPJ were enriched in the samples pulled down from MEC-1 (Fig. 3*B*). Since the BJAB cells demonstrated higher *CD22* transcription than the MEC-1 cells (Fig. 2*B*), the transcription factors enriched in the BJAB samples are more likely to be positive regulators of *CD22*, while the transcription factors enriched in the MEC-1 samples could serve as transcription repressors for *CD22*.

We found that the putative GC-box (encompassing the conserved GGxGxGG motif) on the *CD22* promoter is critical for its transactivation (Fig. 2, *D* and *E*). Indeed, we observed that many transcription factors lost their binding to the *CD22* promoter with the mutated GC-box (Fig. 3*C*). However, the effect of the mutation of the putative E-box (encompassing the conserved AGA motif) was less pronounced (Fig. 3*D*). The mutations of these motifs had limited effects on the pulldown of the transcription factors in MEC-1 (Fig. S4).

### Validation of transcription factors regulating *CD22* transcription

**Figure 4:**
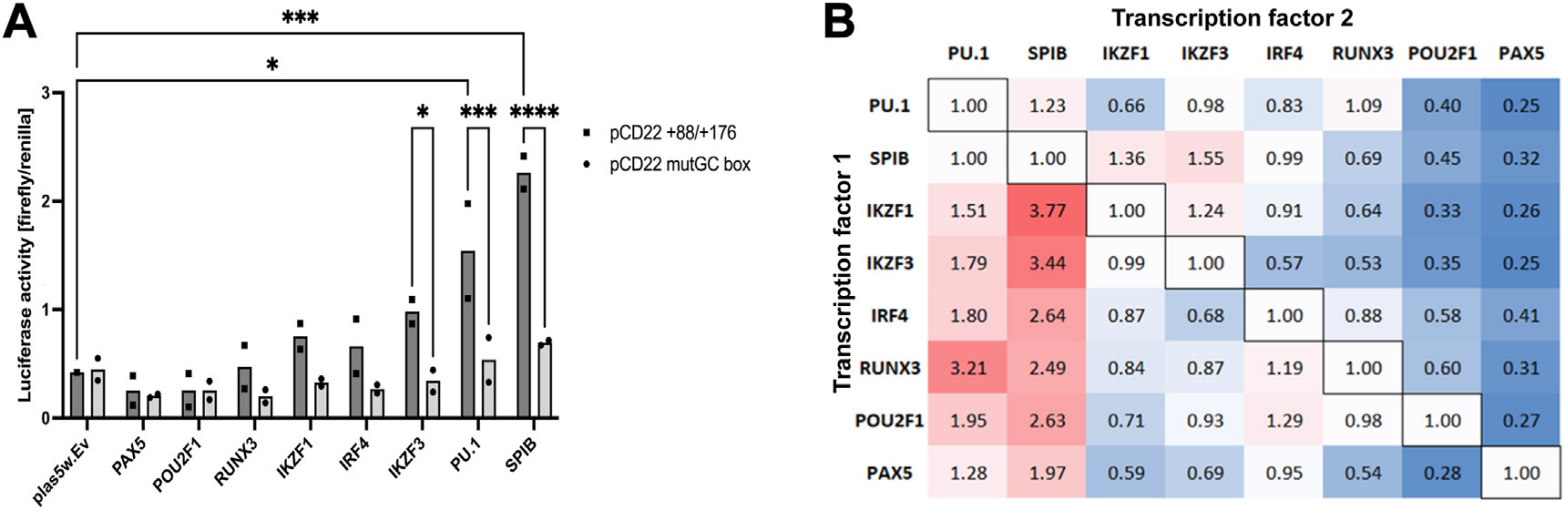
**Luciferase reporter assay by transcription factor reconstitution in the 293T cells.** The expression constructs for transcription factors were cotransfected with the firefly luciferase reporter construct (pGL4.12 with minimal *CD22* promoter) in the 293T cells, and the luciferase reporter assays were conducted. *A*, reporter assay with a single transcription factor. PU.1 and Spi-B positively regulated the reporter expression from the minimal *CD22* promoter. ∗*p* < 0.05, ∗∗∗*p* < 0.001, ∗∗∗∗*p* < 0.0001 (ANOVA with Tukey’s *post hoc* test). *n* = 2 (independent experimental replicates). *B*, reporter assay with the combinations of two transcription factors. The firefly luciferase activity with the single transcription factor (*i.e.*, the cells for which transcription factors 1 and 2 are identical) was used to normalize the values in each row, so that the effects of the co-expressed transcription factors (transcription factor 2) on the reporter expression by the primary transcription factor (transcription factor 1) can be evaluated.

To validate our findings from the proteomics analysis, we selected several identified transcription factors relevant to the B cells or CLL based on the literature and tested their impact on the reporter gene expression under the minimal *CD22* promoter in the 293T cells by cotransfection. We found a strong activation of the reporter expression by PU.1 and Spi-B (the protein products of *SPI1* and *SPIB*, respectively) and a moderate activation by Ikaros and Aiolos (the protein products of *IKZF1* and *IKZF3*, respectively), whereas Pax-5 and Oct-1 (the protein product of *PAX5* and *POU2F1*) may negatively regulate *CD22* transcription (Fig. 4*A*). We also tested the combination of these transcription factors, finding that Spi-B may synergize with Ikaros and Aiolos, whereas Pax-5 and Oct-1 suppressed the reporter expression even in the presence of transcriptional activators (Fig. 4*B*).

### Regulation of *CD22* transcription by PU.1, Spi-B, and IRF4

**Figure 5:**
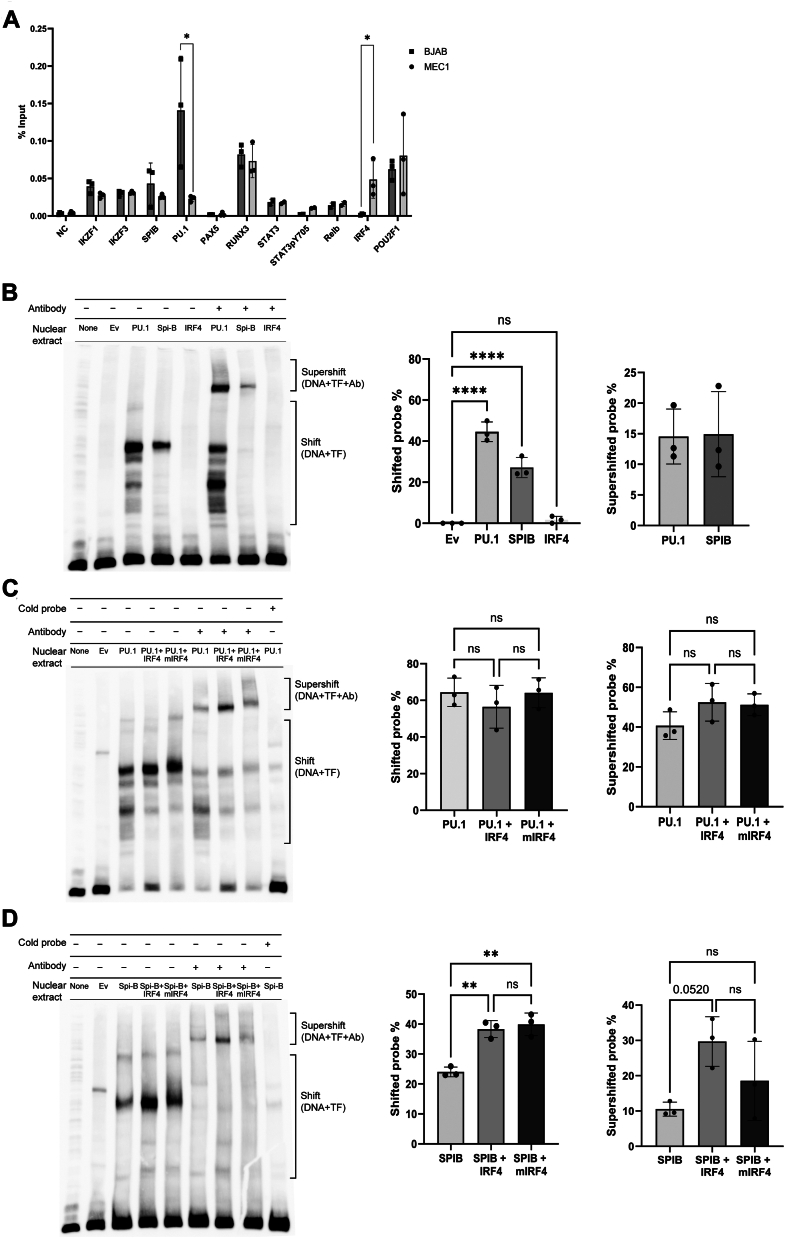
**Regulation of *CD22* transcription by PU.1, Spi-B, and IRF4.***A*, ChIP-qPCR assay. The chromatin DNA–protein complex was prepared from the formalin-fixed BJAB cells by micrococcal nuclease digestion and sonication and subjected to chromatin immunoprecipitation with indicated antibodies. The recovered DNA was purified and subjected to quantitative PCR with a primer set encompassing the minimal *CD22* promoter. The amount of immunoprecipitated DNA was normalized to that of the input. The amount of PU.1 associated with the *CD22* promoter was more abundant in BJAB, whereas that of IRF4 was more abundant in MEC-1. ∗*p* < 0.05 (Student’s *t* test). *n* = 3 (independent experimental replicates). *B*–*D*, electrophoretic mobility shift assays (EMSA) using a DNA probe corresponding to minimal *CD22* promoter and nuclear extracts of 293T cells expressing transcription factor(s). Intensities of bands in the areas labeled “shift” (probe + transcription factor (TF)) and “supershift” (probe + TF + antibody (Ab)) were quantified by densitometry and normalized by the total band intensity of each lane. *B*, binding of PU.1, SpiB, and IRF4 to the minimal *CD22* promoter. PU.1 and Spi-B bound directly to the promoter, whereas IRF4 did not. *C* and *D*, the effect of IRF4 on (*C*) PU.1 and (*D*) Spi-B binding to the minimal *CD22* promoter. IRF4 did not enhance the binding of PU.1 to the minimal *CD22* promoter, whereas it enhanced Spi-B binding. The effect of IRF4 Asp117His mutation (mIRF4; deficient in PU.1/Spi-B association) was not obvious. ∗∗*p* < 0.01, ∗∗∗∗*p* < 0.0001, ns: *p* > 0.2; *p* value is indicated when 0.2 > *p* > 0.05 (ANOVA with Tukey’s *post hoc* test). *n* = 3 (independent experimental replicates).

We performed ChIP-qPCR analysis using the BJAB and MEC-1 cell lines, with a primer set that amplifies the *CD22* minimal promoter region. We found that the occupancy of the *CD22* promoter by PU.1 was higher in BJAB, whereas that by IRF4 was higher in MEC-1 (Fig. 5*A*).

PU.1 and Spi-B belong to the ETS transcription factor family and are involved in B cell development and functional regulation (34, 35, 36, 37). IRF4 is required for the normal development of lymphocytes (38) and is also implicated in the pathology of CLL (39, 40, 41, 42). It is recruited to a composite motif called the ETS-IRF composite element (EICE; consensus: 5′-GGAANNGAAA-3′ (N: any nucleotide) (43)) that is occupied by PU.1 (44, 45) or Spi-B (46). We thus tested whether PU.1, Spi-B, and/or IRF4 could directly bind to the minimal *CD22* promoter using electrophoretic mobility shift assay (EMSA). A biotin-labeled DNA probe corresponding to the minimal *CD22* promoter was mixed with the nuclear extract of the 293T cells that overexpressed one of the transcription factors and separated by PAGE. As shown in Figure 5*B*, PU.1 and Spi-B could bind directly to the minimal *CD22* promoter, whereas IRF4 could not. Using the nuclear extracts of the 293T cells co-expressing IRF4 and PU.1 or Spi-B, we then tested IRF4’s effects on the promoter binding of PU.1 and Spi-B. As shown in Figure 5, *C* and *D*, the binding of PU.1 to the *CD22* minimal promoter was not enhanced by IRF4, whereas that of Spi-B was enhanced. While the Asp117 of IRF4 is considered essential for its heterodimerization with PU.1 or Spi-B (47), the mutation of this residue did not significantly affect the enhanced binding of Spi-B to the minimal *CD22* promoter in the presence of IRF4 (Fig. 5*D*). This may be because the *CD22* minimal promoter may lack a canonical EICE. We also tested the effects of IRF4 on the *CD22* transactivation by PU.1 and Spi-B by reporter assay in the 293T cells, which revealed that IRF4 suppresses reporter transactivation by PU.1, whereas it does not have a strong effect on that by Spi-B (Fig. S5).

**Figure 6:**
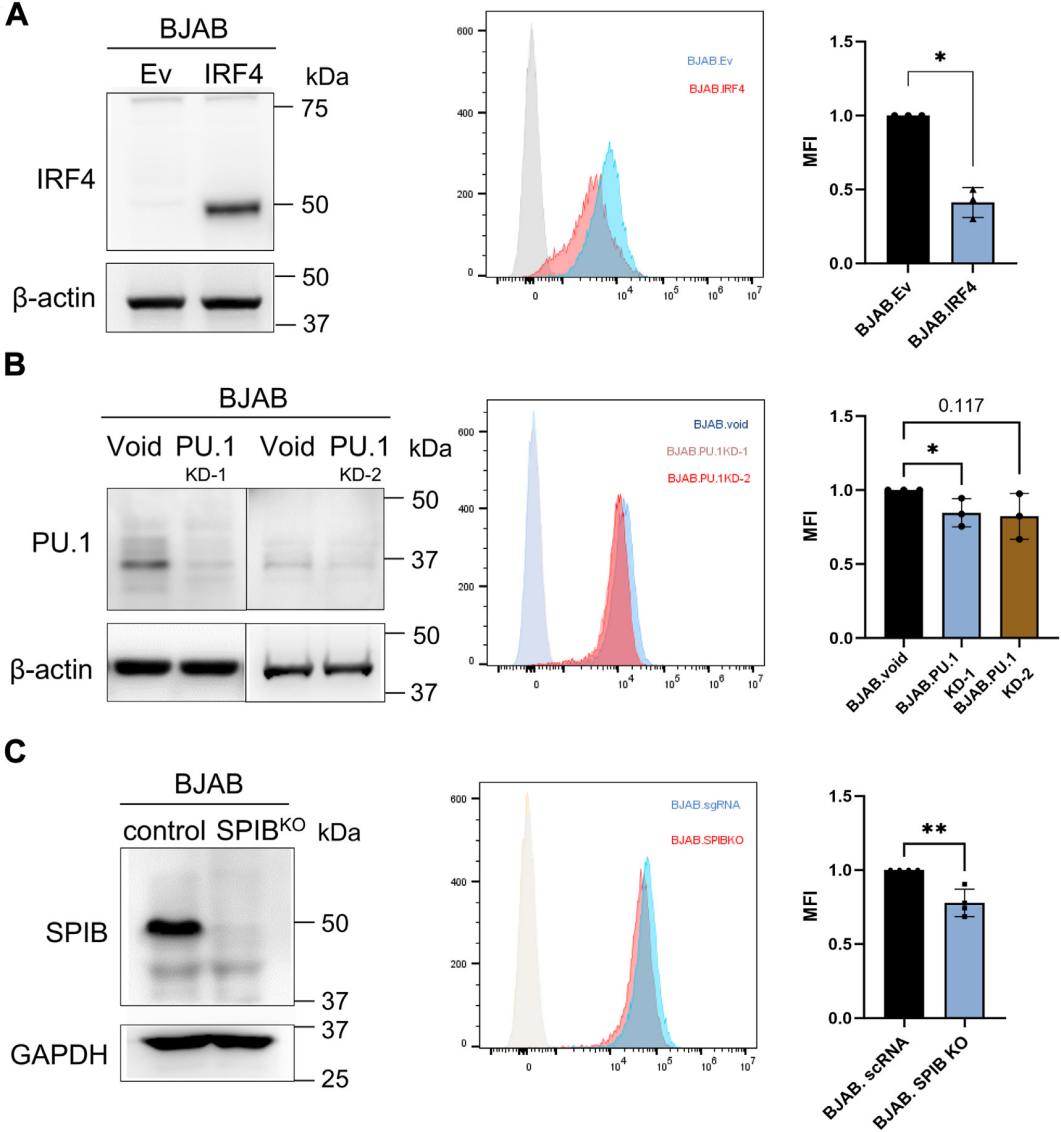
**Effects of transcription factor overexpression/knockdown/knockout in the BJAB cells on CD22 expression.** Effects of (*A*) IRF4 overexpression, (*B*) SPI1 (PU.1) knockdown, (*C*) SPIB KO on CD22 expression, as analyzed with flow cytometry. IRF4 overexpression suppressed CD22 protein expression, whereas knockdown/knockout of PU.1, Spi-B suppressed the expression. ∗*p* < 0.05, ∗∗*p* < 0.01 (Student’s *t* test). *n* = 3 (experimental replicates).

To verify these findings in live cells, we manipulated these genes in the BJAB cell line. The overexpression of IRF4, the knockdown of PU.1, and the knockout of Spi-B in the BJAB cells all significantly reduced the expression of CD22 protein, thereby confirming these proteins’ roles in regulating CD22 expression (Fig. 6, *A*–*C*). However, the effects of PU.1 knockdown and Spi-B knockout on the expression of CD22 protein were modest (∼20% reduction). In the ICGC CLL RNA-Seq dataset, the *CD22* transcript level was positively correlated with those of *SPI1* and *SPIB*, whereas it was negatively correlated with that of *IRF4* (Fig. S6). These results thus corroborate the involvement of these transcription factors in *CD22* transcriptional regulation and its downregulation in CLL B cells.

## Discussion

Although the downregulation of CD22 protein and mRNA in CLL B cells has been reported in the literature, the mechanism underlying the downregulation has been unclear. In this study, we defined the minimal promoter of the human *CD22* gene by reporter assay (Fig. 2) and identified the proteins binding to the *CD22* promoter region by DNA pulldown-proteomics analysis (Fig. 3). Among the proteins that bind to the *CD22* promoter, we found that PU.1 and Spi-B positively regulate *CD22* expression, while IRF4 negatively regulates it (Figs. 4 and 6). We conducted a bioinformatic analysis of a transcriptome dataset of Spanish CLL patients and found that the *CD22* mRNA level is positively associated with those of *SPI1* and *SPIB* (encoding PU.1 and Spi-B) and is negatively associated with that of *IRF4* (Fig. S6). Moreover, we found that a high *CD22* mRNA level is associated with a better prognosis for patients with mCLL (Fig. 1*C*). Our study thus illustrates the basic mechanism of *CD22* transcriptional regulation, which may be relevant to the downregulation of CD22 protein in CLL B cells.

Our finding that IRF4 suppresses the CD22 expression is intriguing, as IRF4 has been implicated in the pathogenesis of CLL by genome-wide association studies (39, 48, 49) and driver mutation studies (27, 40, 50, 51). However, the associations implied by these two lines of studies appear to be incongruent: while the genome-wide association study results imply that the allele conferring the reduced expression of IRF4 is a risk allele (39), the mutational studies imply that the gain-of-function mutation (L116R) (52) and copy number gain (51) are drivers of CLL. Although the association between high IRF4 expression and better prognosis of patients with CLL has been reported (53) and studies using mouse models of CLL with IRF4 deficiency support the role of IRF4 in suppressing CLL pathogenesis (41, 42, 54), the direction of association between IRF4 expression and CLL prognosis remains inconclusive and may even differ between mCLL and uCLL (Fig. S7). Further investigations are required to understand the mechanism underpinning the associations between IRF4 and CLL. Likewise, the mechanism by which *CD22* transcription is negatively regulated by IRF4 remains unclear. Our ChIP-PCR data (Fig. 5*A*) indicates that IRF4 is associated with the endogenous *CD22* promoter (or the DNA segment nearby) in MEC-1 cells. However, our EMSA data (Fig. 5*B*) indicates that IRF4 does not directly bind to the minimal promoter of *CD22* on its own, whereas it enhances the binding of Spi-B to the minimal promoter. These results imply that the recruitment of IRF4 to the *CD22* promoter may depend on PU.1 or Spi-B but may not involve direct DNA binding. IRF4 cooperatively upregulates the transcription of several key genes involved in B cell development and differentiation with PU.1 (44, 45) or Spi-B (46), and the composite sequence element called EICE is implicated in these processes. However, the immediate upstream of the human *CD22* gene (*i.e.*, minimal promoter) appears to lack a canonical EICE. The precise mechanism by which IRF4 represses *CD22* transcription thus demands further investigation.

Our bioinformatic analysis revealed that a high level of *CD22* mRNA is associated with a better prognosis in patients with mCLL (Fig. 1*C*). CD22 may be thus useful as a marker (or a part of marker sets) for patient stratification. In addition, our experiments using cell lines implied that the IRF4 suppression may upregulate CD22, thereby altering the course of CLL. The upregulation of CD22 may influence the proliferation of CLL B cells by suppressing the BCR signaling. In addition, it may provide a target for immunotherapy. Since the inefficiency of CD22-targeting drugs against CLL may be explained by the low expression of CD22 protein on CLL B cells (20, 21), the combination of IRF4 inhibition and CD22-targeted therapy (*e.g.*, immunotherapy using antibody or chimeric antigen receptor) may be worth exploring. Despite our repeated attempts to knockout or knockdown IRF4 in CLL cell lines, we failed to obtain an IRF4-suppressed population owing to extensive cell death.

Our study has some limitations. First, owing to the limited number of CLL patients in Taiwan, we could not correlate the expression level of CD22 (transcript or protein) on B cells with the prognosis of Taiwanese CLL patients. Nevertheless, the *CD22* transcript abundance appears to be a good surrogate of the CD22 protein level (Fig. 1*B*), and the positive correlation between high *CD22* transcript levels and overall survival of Spanish mCLL patients (Fig. 1*C*) warrants future investigation of the association between CD22 protein expression level and prognosis, which may be extended to encompass other candidate prognosis markers by high-dimensional cytometry. Second, while our experiments using cell lines showed that PU.1 and Spi-B promote, whereas IRF4 represses, CD22 transcription (Fig. 6), it did not exclude the possibility that other transcription factors play a role in *CD22* transcriptional regulation. IRF4 did not appear to bind directly to the minimal promoter of *CD22* (Fig. 6), leaving an unanswered question regarding the mechanism by which IRF4 represses *CD22* transcription. Regardless of these limitations, our study is the first to clearly define the minimal promoter of the human *CD22* gene and identify several of its transcriptional regulators. This knowledge will facilitate the development of a strategy to target CD22 for CLL therapy.

## Experimental procedures

### Patient samples

Peripheral blood from patients with CLL and healthy donors were obtained at the National Taiwan University Hospital and the Taipei Blood Center, respectively. Informed consent was obtained from each donor. The institutional review boards of the National Taiwan University Hospital and Academia Sinica approved the study. The studies in this work abide by the Declaration of Helsinki principles.

B cells were isolated from the peripheral blood of the donors using Ficoll-Paque PLUS (17-1440-03, Cytiva), which was followed by affinity purification with CD19 MicroBeads (130-050-301, Miltenyi Biotec). The cells were then either used immediately for flow cytometry or stored frozen at −80 °C until being used for RNA-Seq.

### Flow cytometry of human peripheral blood B cells

The freshly prepared B cells (1 × 10^6^ cells) were stained with an allophycocyanin-conjugated anti-CD22 antibody (302510, Biolegend) and analyzed using a flow cytometer (BD FACSCanto II, BD Biosciences). We processed the data with FlowJo ver.10 (BD Biosciences).

### RNA-Seq analysis of B cells from patients with CLL

Total RNA was prepared from 5 × 10^6^ cells using the RNeasy Mini Kit (74104, Qiagen) and subjected to library preparation using the KAPA mRNA HyperPrep Kit (Roche). Next-generation sequencing (20 million paired-end (2 × 150 bp) sequencing per sample) with NovaSeq 6000 (Illumina) was performed at BioTools. The raw data were mapped and normalized, and the transcripts per million were calculated for each gene using HISAT2 (55). The dataset was deposited in the NCBI GEO database (accession number: GSE273421).

### Cell culture

All the cells were cultured in humidified incubators at 37 °C with 5% CO_2_. BJAB (ACC-757, DSMZ–German Collection of Microorganisms and Cell Cultures), a Burkitt lymphoma cell line, was cultured in RPMI 1640 (11875093, Gibco) supplemented with 20% fetal bovine serum (FBS; A3160502, Gibco), 100 U/ml penicillin, and 100 μg/ml streptomycin (Pen/Strep; 15140122, Gibco). MEC-1 (ACC-497, DSMZ), a CLL cell line, was maintained in IMDM (12440053, Gibco) supplemented with 10% FBS and Pen/Strep. JVM-3 (ACC-18, DSMZ), a CLL cell line, was maintained in RPMI 1640 (11875093, Gibco) supplemented with 10% FBS and Pen/Strep. The 293T cells (CRL-3216, ATCC) were cultured in Dulbecco’s modified Eagle’s medium (11965092, Gibco) supplemented with 10% FBS and Pen/Strep. We ensured that all the cell lines we used were free from *mycoplasma* contamination by testing them using the EZ-PCR *Mycoplasma* Detection Kit (Biological Industries USA, 20-700-20).

### Quantitative PCR analysis of *CD22* transcripts in B cell lines

Total RNA was prepared from the B cell lines using the RNeasy Plus Mini Kit (74134, Qiagen), reverse-transcribed with SuperScript III First-Strand Synthesis SuperMix (18080400, Thermo Fisher Scientific), and subjected to quantitative PCR analysis using the TaqMan reagent set (Hs00998488; Thermo Fisher Scientific) and the FastStart Universal Probe Master (Rox) (04914058001, Roche) with StepOnePlus RT-PCR Systems (Thermo Fisher Scientific). PGK1 was used for normalization (Hs99999906; Thermo Fisher Scientific).

### Preparation of luciferase reporter constructs

The luciferase reporter constructs, which contain the genomic segment encompassing the 1.5 kb upstream and 0.4 kb downstream of the human *CD22* TSS (−1499 to +415 of the TSS as defined by Wilson *et al.* (25)) and its sub-sections, were prepared by performing the PCR amplification of the genomic DNA from the JVM-3 cell line and cloning them to the KpnI-XhoI sites of pGL4.12[luc2CP] (E667A, Promega). The primer sequences are provided in the Table S1.

### Preparation of minimal *CD22* promoter construct with mutations at the GC-box or E-box

The minimal CD22 promoter (+88 to +176 from the TSS) constructs with mutations in the putative GC-box (+107 to +111) or E-box (+171 to +176) were prepared using site-directed mutagenesis (56). The primer sequences are provided in the Table S1.

### Luciferase reporter assay in B cell lines

Cells (1 × 10^7^) were harvested by centrifugation, washed once with ice-cold PBS, and resuspended in 450 μl of ice-cold culture medium without FBS. A reporter construct (20 μg; with the human *CD22* promoter region/subregion inserted upstream of the firefly luciferase cDNA on pGL4.12[luc2CP] vector) and a control plasmid (2 μg; pGL4.74[hRluc/TK] encoding the *Renilla* luciferase cDNA under the CMV promoter (E692A, Promega)) were mixed with the cells and transferred into a prechilled 0.4 cm cuvette (1652088, Bio-Rad). After a 5 min incubation on ice, electroporation was performed at 250 V and 950 μF using an electroporator (Gene Pulser Xcell, Bio-Rad). The cuvette was kept on ice for 5 min, and then the content was transferred to a 10 cm dish in which 9 ml of RPMI 1640 containing 20% FBS was added and was cultured overnight. The next day, we collected the cells, washed them with ice-cold PBS, and suspended them in 300 μl PBS. We analyzed the luciferase activities using the Dual-Glo Luciferase Assay System (E2920, Promega). Briefly, we transferred the cell suspensions (100 μl) to a 96-well black plate, to which we added a 100 μl/well of Dual-Glo Luciferase Assay Reagent (for firefly luciferase activity). The plate was incubated on a shaker for 10 min, following which we performed chemiluminescence quantification using a plate reader (SpectraMax Paradigm, Molecular Devices, equipped with a Glow Luminescence (LUM) Detection Cartridge). After we quantified the firefly luciferase activity, a 100 μl/well of Dual-Glo Stop & Glo Reagent (for the *Renilla* luciferase activity) was added, and the plate was incubated on a shaker for 10 min, followed by chemiluminescence quantification with the plate reader. We normalized the firefly luciferase activity using the *Renilla* luciferase activity in the same well.

### DNA pulldown – proteomics

#### Experimental design

We adopted a previously described workflow (30, 31). A 5′-biotinylated DNA probe corresponding to the minimal *CD22* promoter (+88 to +178 from the TSS) was used as a bait to capture the transcriptional regulators from the nuclear extracts of the cell lines in which CD22 protein was expressed at a high (BJAB) or a low (MEC-1) level. To facilitate the subsequent elution of bound proteins, we added an EcoRI recognition site near the 5′ end of the DNA. We also used two probes mutated at putative GC-box (+107 to +111 from the TSS) and E-box (+171 to +176 from the TSS) motifs. The same experiment was repeated twice (*n* = 2, independent experimental replications).

#### Preparation of biotinylated DNA probe

Biotinylated DNA probes were generated by PCR using a biotinylated “Forward_CD22_probe” primer and a “Reverse_CD22_probe” primer (see Table S1). We purified the PCR product with 1.5% agarose gel electrophoresis and extraction (QIAquick Gel Extraction Kit, Qiagen).

#### Nuclear extract preparation

We collected the cells (∼2–3 × 10^8^) in a 50 ml tube, washed them with ice-cold PBS, suspended them in 8 ml of buffer A (10 mM Hepes, pH 7.9, 10 mM KCl, 0.1 mM EDTA, 1 mM DTT, and protease inhibitors), and incubated them on ice for 30 min. We further supplemented the mixture with IGEPAL CA-630 (500 μl of 10% (v/v) solution in water), which was vortexed for 10 s, incubated for 5 min on ice, vortexed again for 10 s, and centrifuged at 15,000*g* for 30 min at 4 °C. The pellet (containing nuclei) was resuspended in 2 ml of buffer C (20 mM Hepes, pH 7.9, 400 mM NaCl, 1 mM EDTA, 1 mM DTT, 10% glycerol, and protease inhibitors), and the nuclear proteins were released by sonication. The sonication was performed at level 3, with 10 s of pulses followed by 20 s of rest on ice, repeated 15 times (MICROSON XL2000, Misonix). The lysate was centrifuged at 15,000*g* for 30 min at 4 °C, and the supernatant was then collected. We determined the protein concentration using the Pierce BCA Protein Assay Kit (23227, Thermo Fisher Scientific).

#### DNA pulldown

The biotinylated DNA probe (40 pmol) was immobilized onto the Dynabeads Streptavidin MyOne (0.75 mg; 65002, Thermo Fisher Scientific) by incubating it at room temperature for 1 h with rotation. We washed the beads three times with a washing buffer (PBS containing 0.01% Tween 20). In parallel, we incubated 2 mg of nuclear extract with 1.5 volumes of binding buffer (4 mM Hepes, pH 7.5, 120 mM KCl, 8% glycerol, 2 μM DTT, 0.166 μg/μl salmon sperm DNA) on ice for 15 min. Then, we mixed the nuclear extract in the binding buffer with the DNA–Dynabeads complex and incubated it at room temperature for 1 h with rotation. The beads were serially washed once with the binding buffer, three times with the washing buffer, and once with the EcoRI buffer (100 mM Tris–HCl, pH 7.5, 50 mM NaCl, and 10 mM MgCl_2_), and then incubated with EcoRI (1000 U; R0101S, New England Biolabs) in 30 μl of the EcoRI buffer at 37 °C for 25 min with resuspension every 5 min. The supernatant was then collected, mixed with an equal volume of 2 × SDS-PAGE sample buffer, and boiled at 100 °C for 5 min.

#### Front-end sample processing

We subjected the samples (30 μl) to a short SDS-PAGE (10% separation gel) and stained the gel with the Bio-Safe Coomassie Stain (1610786, Bio-Rad). Afterward, we excised the gel bands, dissected them into ∼1 mm^3^ cubes, de-stained them using 50% acetonitrile (ACN) in 25 mM ammonium bicarbonate (ABC), and reduced the proteins therein by incubating them in 100 μl of 50 mM dithioerythritol in 25 mM ABC at 37 °C for 1 hour. The buffer was replaced with 100 μl of 100 mM iodoacetamide in 25 mM ABC, and the gel pieces were incubated at room temperature in the dark for 1 h. We washed the gel pieces four times with 200 μl of 50% ACN in 25 mM ABC, incubated them at room temperature for 15 min, dehydrated them with 100% ACN, and completely dried them using SpeedVac. We then rehydrated and digested the samples with LysC (1/50 of the total protein quantity) in 25 mM ABC at 37 °C for 2 h, followed by overnight digestion with trypsin (1/50 of the total protein quantity). The reaction was stopped with 50 μl of 50% ACN in 5% TFA, followed by sonication and centrifugation. The supernatant was transferred to a new tube, and the peptide extraction by sonication and centrifugation was repeated twice. The combined supernatant was dried using SpeedVac, dissolved in 10 μl of 0.1% formic acid, and desalted with ZipTip with C18 resin (EMD Millipore).

#### Mass spectrometry (MS) data acquisition

We acquired the MS data at the Academia Sinica Common Mass Spectrometry Facility using NanoLC-nanoESI-MS/MS with a nanoAcquity system (Waters) connected to the Orbitrap Elite hybrid mass spectrometer (Thermo Fisher Scientific) and equipped with a PicoView nanospray interface (New Objective). The peptide mixtures were loaded onto a C18 BEH column (75 μm ID × 25 cm length, Waters) packed with 1.7 μm particles with a pore size of 130 Å and separated by a segmented gradient in 90 min with a flow rate of 300 nl/min and a column temperature of 35 °C. The mass spectrometer was operated in the data-dependent mode. Briefly, survey full scan MS spectra were acquired in the orbitrap (m/z 350–1600) with the resolution set to 120K at m/z 400 and automatic gain control target at 10^6^. The 20 most intense ions were sequentially isolated for CID MS/MS fragmentation and detection in the linear ion trap (automatic gain control target at 10,000) with previously selected ions dynamically excluded for 60 s. We excluded singly charged ions and those with unrecognized charge states. We deposited the proteomics dataset in the PRoteomics IDEntifications (PRIDE) database (accession number: PXD054553) through ProteomeXchange (57).

#### MS data processing

We analyzed the raw datasets of all the samples with the MaxQuant software package (version 1.6.16.0) (32), which includes the Andromeda peptide search engine (58). We used the human protein FASTA files downloaded from UniProt in July 2018. Carbamidomethylation (C) was set as a fixed modification, and oxidation (M) was set as a variable modification. LysC and trypsin were selected as proteases. The false discovery rate was set to 1% in all the fields. The second peptide and the match between run options were enabled. The relative label-free quantification of proteins and classic normalization options were selected.

#### Bioinformatic analysis

We performed further data processing on the output files for the protein and peptide quantitation obtained from MaxQuant using Perseus software (version 1.6.12.0) (59). The label-free quantification results were used for further data analysis. The hits identified as potential contaminants and those identified by the reverse database were filtered out, and the proteins identified with three or more peptides and with at least one unique peptide were filtered in. Protein abundance was normalized by the total peak areas in the same sample (Table S2), and the normalized abundance value was log2-transformed. Unpaired Student’s T-tests were performed between BJAB *versus* MEC-1 nuclear proteins extracted with the WT probe, as well as between the WT *versus* mutated probes. The proteins whose abundance was at least twice as much in one of the samples being compared and with a *p* value < 0.05 were considered significant.

### ChIP-qPCR assay

To crosslink proteins and DNA, we treated 4 × 10^6^ cells with 1% formaldehyde at room temperature for 10 min. The excess formaldehyde was quenched by adding glycine (final concentration: 125 μM) and incubating it for 5 min. Chromatin immunoprecipitation was performed using the Pierce Magnetic ChIP Kit (26157, Thermo Fisher Scientific). In brief, the cells were lysed, the chromatin was digested with micrococcal nuclease and then sonicated on ice to further fragment the chromatin. Immunoprecipitation was performed using 10 μg of antibodies specific to the candidate transcription regulators and 25 μg of the crosslinked DNA with overnight incubation. The antibody–chromatin complex was captured using the Protein A/G magnetic beads, and the cross-links were then reversed. The DNA was recovered and subjected to quantitative PCR analyses using the primers amplifying the minimal *CD22* promoter region (“Forward_pCD22” and “Reverse_pCD22”; see Table S1) and a quenched probe (“qPCR_probe_pCD22” labeled with 5′ FAM and 3′ NFQ). The relative abundance of the precipitated target region was normalized using input control.

### Cotransfection of reporter and transcription factor constructs in 293T cells

We transfected the 293T cells (80%–90% confluent) in 24-well plates with a mixture of 100 ng pGL4.12[luc2CP] vector containing the *CD22* minimal promoter, 10 ng of pGL4.74[hRluc/TK], 400 ng of plasmid(s) encoding transcription factor(s), and 1.5 μl Lipofectamine 2000 (11668019, Thermo Fisher Scientific). At 24 h post-transfection, we analyzed firefly and *Renilla* luciferase activities using the Dual-Glo Luciferase Assay System (Promega), as described above.

### Electrophoretic mobility shift assay

#### Transient transfection in the 293T cells

We transiently transfected the 293T cells (80%-90% confluent) in a 6 cm dish with 6 μg of pLAS5w.PU.1.puro, pLAS5w.SPIB.puro, or pLAS5w.IRF4.puro plasmid and 24 μl of Lipofectamine 2000. The medium was replaced with fresh culture medium after 24 h, and we collected the cells the next day for nuclear extraction.

#### Nuclear extraction

We collected the transfected cells by centrifugation at 500*g* for 3 min at 4 °C, and they were washed with 10 ml of ice-cold PBS. The pellets were then resuspended in 800 μl of buffer A (10 mM Hepes, pH 7.9, 10 mM KCl, 0.1 mM EDTA, 1 mM DTT, and protease inhibitors) and incubated on ice for 15 min. IGEPAL CA-630 (50 μl of 10% (v/v) solution in water) was added to each sample and incubated for 1 min on ice, followed by vigorous vortexing for 5 s and centrifugation at 16,000*g* for 5 min at 4 °C. The pellet was washed with 800 μl of buffer A and suspended in 200 μl of buffer C (20 mM Hepes, pH 7.9, 400 mM NaCl, 1 mM EDTA, 1 mM DTT 10% glycerol, and protease inhibitors), followed by four rounds of vigorous vortexing (15 s) and incubation on ice (10 min). After the fifth vortexing, we centrifuged the samples at 16,000*g* for 10 min at 4 °C and transferred the supernatant containing nuclear proteins to a new 1.5 ml tube. The protein concentration was then determined using the BCA assay.

#### Electrophoretic mobility shift assay

We used the LightShift Chemiluminescent EMSA Kit (20148, Thermo Fisher Scientific) for the assay. The same biotinylated probe (representing the minimal *CD22* promoter) used for the affinity purification of nuclear proteins above was used along with a nonbiotinylated probe. The biotinylated DNA probe was incubated with 5 μg of nuclear extract in the 1 × binding buffer containing 50 ng/μl Poly(dI∗dC), 2.5% glycerol, 0.05% IGEPAL CA-630, 5 mM MgCl_2_, 50 mM KCl, and 10 mM EDTA for 30 min on ice. The complete binding samples were loaded onto a well of 6% native polyacrylamide gel in a 0.5 × Tris-Borate-EDTA buffer system and electrophoresed at 100V for 4 h on ice. The DNA–protein complex was then transferred onto a Nylon membrane (NEF988001PK, Revvity) at 380 mA for 30 min on ice, followed by crosslinking using a UV transilluminator (312 nm) for 10 min. The membrane was blocked with 20 ml of blocking buffer for 15 min; the biotinylated DNA was then detected using the streptavidin-horseradish peroxidase conjugate, and a digital image was captured with an ImageQuant LAS 4000 mini luminescent imaging analyzer (GE Healthcare). Intensities of bands were quantified by ImageJ (version 1.53).

### Genetic manipulation of B cell lines

The construct preparation, lentivirus production, and lentiviral transduction are described in the Supplementary Methods section.

#### Over-expression of IRF4

The BJAB cells were lentivirally transduced with a transfer vector encoding an epitope-tagged IRF4. After drug selection, the cells were characterized without further cloning.

#### Knockdown of SPI1

The BJAB cells were lentivirally transduced with a transfer vector encoding a shRNA targeting *SPI1* (TRCN0000417534 or TRCN0000426240, obtained from the RNA Technology Platform and Gene Manipulation Core, Academia Sinica). After drug selection, the cells were characterized without further cloning.

#### Knockout of SPIB

The BJAB cells that stably express Cas9 were prepared using lentiviral transduction with a transfer vector encoding an HA-tagged Cas9 (p5w.Cas9.Pbsd, obtained from the RNA Technology Platform and Gene Manipulation Core, Academia Sinica). After drug selection, single-cell clones were established by limiting dilution. The clones were lentivirally transduced with a transfer vector encoding single-guide RNA (5′-AGGTCATAGAAGACGCCATC-3′; prepared by the RNA Technology Platform and Gene Manipulation Core, Academia Sinica). After drug selection, single-cell clones were established by limiting dilution and characterized.

### Western blotting

We used the RIPA lysis buffer (50 mM Tris–HCl, 150 mM NaCl, 1% IGEPAL CA-630, 0.5% sodium deoxycholate, and 0.1% SDS) supplemented with a protease inhibitor cocktail (cOmplete Tablets, Roche, 04693159001) to extract proteins from cell lines. We then quantified the proteins using the BCA Protein Assay Kit (23225, Pierce). We mixed the cell lysates with SDS-PAGE sample buffer (Bio-Rad) and boiled them. The proteins therein were then separated using 10% SDS separation gel (TGX Stain-Free FastCast Acrylamide; 1610183, Bio-Rad) and transferred onto the PVDF membrane (10600023, Cytiva) using a semi-dry transfer system (Bio-Rad). The membrane was blocked by 3% BSA in 20 mM Tris–Cl pH 7.5, 150 mM NaCl, and 0.1% Tween-20 (TBST) at room temperature for 1 h, followed by overnight incubation with the primary antibody at 4 °C. We washed the membrane three times with TBST for 10 min and incubated it with an appropriate peroxidase-conjugated secondary antibody for 1 h at room temperature. The membrane was then washed three times with TBST for 10 min and incubated with an ECL reagent (Western Lightning ECL Pro; NEL122001EA, Revvity). The chemiluminescence signals were imaged using the ImageQuant LAS4000mini luminescent imaging analyzer (GE Healthcare). The antibodies used in this study are listed in Table S3.

### Bioinformatic analysis of the transcriptomic dataset of Spanish patients with CLL

The normalized RNA-Seq dataset of the B cells from the Spanish patients with CLL (disease type labeled as CLL or SLL) and the associated metadata (27) were acquired from the International Cancer Genome Consortium website. We stratified the patients based on the *IGHV* mutation status (mutated *versus* unmutated), and the cutp function in survMisc (60), an R package, was used to find the optimal cutoff for dichotomizing the *CD22*^high^ and *CD22*^low^ patients for performing the association analysis in the Cox proportional hazards model. The Kaplan-Meier plots, indicating the overall survival of *CD22*^high^ and *CD22*^low^ patients in each group (*i.e.*, *IGHV*-mutated *versus* unmutated), were generated with the survival package for R (61).

### Statistical analyses

Statistical analyses were performed with Prism (version 10, GraphPad) or R. The statistical test applied for each dataset is described in the main text or figure legend.

## Data availability

Transcriptomic dataset was deposited in the NCBI GEO database (accession number: GSE273421). Proteomic dataset was deposited in the PRoteomics IDEntifications (PRIDE) database (accession number: PXD054553). Other data are contained within the manuscript.

## Supporting information

This article contains supporting information.

## Conflicts of interest

The authors declare that they have no conflicts of interests with the contents of this article.

## Supporting information

Supporting info.pdf

## References

[1] Kipps T.J., Stevenson F.K., Wu C.J., Croce C.M., Packham G., Wierda W.G. (2017). Chronic lymphocytic leukaemia. Nat. Rev. Dis. Primers.

[2] Bosch F., Dalla-Favera R. (2019). Chronic lymphocytic leukaemia: from genetics to treatment. Nat. Rev. Clin. Oncol..

[3] Damle R.N., Wasil T., Fais F., Ghiotto F., Valetto A., Allen S.L. (1999). Ig V gene mutation status and CD38 expression as novel prognostic indicators in chronic lymphocytic. Leuk. Blood.

[4] Hamblin T.J., Davis Z., Gardiner A., Oscier D.G., Stevenson F.K. (1999). Unmutated Ig V(H) genes are associated with a more aggressive form of chronic lymphocytic leukemia. Blood.

[5] Burger J.A., Chiorazzi N. (2013). B cell receptor signaling in chronic lymphocytic. Leukemia Trends Immunol..

[6] Burger J.A. (2020). Treatment of chronic lymphocytic leukemia. N. Engl. J. Med..

[7] Hallek M., Al-Sawaf O. (2021). Chronic lymphocytic leukemia: 2022 update on diagnostic and therapeutic procedures. Am. J. Hematol..

[8] Woyach J.A., Johnson A.J. (2015). Targeted therapies in CLL: mechanisms of resistance and strategies for management. Blood.

[9] Fraietta J.A., Lacey S.F., Orlando E.J., Pruteanu-Malinici I., Gohil M., Lundh S. (2018). Determinants of response and resistance to CD19 chimeric antigen receptor (CAR) T cell therapy of chronic lymphocytic leukemia. Nat. Med..

[10] Liu E., Marin D., Banerjee P., Macapinlac H.A., Thompson P., Basar R. (2020). Use of CAR-transduced natural killer cells in CD19-positive lymphoid tumors. N. Engl. J. Med..

[11] Meyer S.J., Linder A.T., Brandl C., Nitschke L. (2018). B cell siglecs-news on signaling and its interplay with ligand binding. Front. Immunol..

[12] Tsubata T. (2018). Ligand recognition determines the role of inhibitory B cell Co-receptors in the regulation of B cell homeostasis and autoimmunity. Front. Immunol..

[13] Tuscano J.M., Riva A., Toscano S.N., Tedder T.F., Kehrl J.H. (1999). CD22 cross-linking generates B-cell antigen receptor-independent signals that activate the JNK/SAPK signaling cascade. Blood.

[14] Macauley M., Paulson J. (2014). Siglecs induce tolerance to cell surface antigens by BIM-dependent deletion of the antigen-reactive B cells. J. Immunol..

[15] Rafei H., Kantarjian H.M., Jabbour E.J. (2019). Recent advances in the treatment of acute lymphoblastic leukemia. Leuk. Lymphoma.

[16] Shah N.N., Sokol L. (2021). Targeting CD22 for the treatment of B-cell malignancies. Immunotargets Ther..

[17] D'Arena G., Musto P., Cascavilla N., Dell'Olio M., Di Renzo N., Carotenuto M. (2000). Quantitative flow cytometry for the differential diagnosis of leukemic B-cell chronic lymphoproliferative disorders. Am. J. Hematol..

[18] Huang J., Fan G., Zhong Y., Gatter K., Braziel R., Gross G. (2005). Diagnostic usefulness of aberrant CD22 expression in differentiating neoplastic cells of B-Cell chronic lymphoproliferative disorders from admixed benign B cells in four-color multiparameter flow. Cytometry Am. J. Clin. Pathol..

[19] Payelle-Brogard B., Dumas G., Magnac C., Lalanne A.I., Dighiero G., Vuillier F. (2006). Abnormal levels of the alpha chain of the CD22 adhesion molecule may account for low CD22 surface expression in chronic lymphocytic. Leuk. Leuk..

[20] Kreitman R., Pastan I. (2011). Antibody fusion proteins: anti-CD22 recombinant immunotoxin moxetumomab pasudotox. Clin. Cancer Res..

[21] Advani R.H., Lebovic D., Chen A., Brunvand M., Goy A., Chang J.E. (2017). Phase I study of the anti-CD22 antibody-drug conjugate pinatuzumab vedotin with/without rituximab in patients with relapsed/refractory B-cell non-hodgkin lymphoma. Clin. Cancer Res..

[22] Psathas J.N., Doonan P.J., Raman P., Freedman B.D., Minn A.J., Thomas-Tikhonenko A. (2013). The Myc-miR-17-92 axis amplifies B-cell receptor signaling via inhibition of ITIM proteins: a novel lymphomagenic feed-forward loop. Blood.

[23] John B., Herrin B., Raman C., Wang Y., Bobbitt K., Brody B. (2003). The B cell coreceptor CD22 associates with AP50, a clathrin-coated pit adapter protein, via tyrosine-dependent interaction. J. Immunol..

[24] Seifert M., Sellmann L., Bloehdorn J., Wein F., Stilgenbauer S., Durig J. (2012). Cellular origin and pathophysiology of chronic lymphocytic leukemia. J. Exp. Med..

[25] Wilson G.L., Najfeld V., Kozlow E., Menniger J., Ward D., Kehrl J.H. (1993). Genomic structure and chromosomal mapping of the human CD22 gene. J. Immunol..

[26] Andersson K.B., Draves K.E., Magaletti D.M., Fujioka S., Holmes K.L., Law C.L. (1996). Characterization of the expression and gene promoter of CD22 in murine B cells. Eur. J. Immunol..

[27] Puente X.S., Bea S., Valdes-Mas R., Villamor N., Gutierrez-Abril J., Martin-Subero J.I. (2015). Non-coding recurrent mutations in chronic lymphocytic leukaemia. Nature.

[28] Ferreira P.G., Jares P., Rico D., Gomez-Lopez G., Martinez-Trillos A., Villamor N. (2014). Transcriptome characterization by RNA sequencing identifies a major molecular and clinical subdivision in chronic lymphocytic leukemia. Genome Res..

[29] Zoonomia Consortium (2020). A comparative genomics multitool for scientific discovery and conservation. Nature.

[30] Mittler G., Butter F., Mann M. (2009). A SILAC-based DNA protein interaction screen that identifies candidate binding proteins to functional DNA elements. Genome Res..

[31] Tacheny A., Michel S., Dieu M., Payen L., Arnould T., Renard P. (2012). Unbiased proteomic analysis of proteins interacting with the HIV-1 5'LTR sequence: role of the transcription factor. Meis Nucleic Acids Res..

[32] Tyanova S., Temu T., Cox J. (2016). The MaxQuant computational platform for mass spectrometry-based shotgun. Proteomics Nat. Protoc..

[33] Thomas P.D., Ebert D., Muruganujan A., Mushayahama T., Albou L.P., Mi H. (2022). PANTHER: Making genome-scale phylogenetics accessible to all. Protein Sci..

[34] Scott E.W., Simon M.C., Anastasi J., Singh H. (1994). Requirement of transcription factor PU.1 in the development of multiple hematopoietic lineages. Science.

[35] McKercher S.R., Torbett B.E., Anderson K.L., Henkel G.W., Vestal D.J., Baribault H. (1996). Targeted disruption of the PU.1 gene results in multiple hematopoietic abnormalities. EMBO J..

[36] Su G.H., Chen H.M., Muthusamy N., Garrett-Sinha L.A., Baunoch D., Tenen D.G. (1997). Defective B cell receptor-mediated responses in mice lacking the Ets protein, Spi-B. EMBO J..

[37] Garrett-Sinha L.A., Su G.H., Rao S., Kabak S., Hao Z., Clark M.R. (1999). PU.1 and Spi-B are required for normal B cell receptor-mediated signal transduction. Immunity.

[38] Mittrucker H.W., Matsuyama T., Grossman A., Kundig T.M., Potter J., Shahinian A. (1997). Requirement for the transcription factor LSIRF/IRF4 for mature B and T lymphocyte function. Science.

[39] Di Bernardo M.C., Crowther-Swanepoel D., Broderick P., Webb E., Sellick G., Wild R. (2008). A genome-wide association study identifies six susceptibility loci for chronic lymphocytic leukemia. Nat. Genet..

[40] Havelange V., Pekarsky Y., Nakamura T., Palamarchuk A., Alder H., Rassenti L. (2011). IRF4 mutations in chronic lymphocytic leukemia. Blood.

[41] Shukla V., Ma S., Hardy R.R., Joshi S.S., Lu R. (2013). A role for IRF4 in the development of CLL. Blood.

[42] Asslaber D., Qi Y., Maeding N., Steiner M., Denk U., Hopner J.P. (2019). B-cell-specific IRF4 deletion accelerates chronic lymphocytic leukemia development by enhanced tumor immune evasion. Blood.

[43] Marecki S., Riendeau C.J., Liang M.D., Fenton M.J. (2001). PU.1 and multiple IFN regulatory factor proteins synergize to mediate transcriptional activation of the human IL-1 beta gene. J. Immunol..

[44] Pongubala J.M., Van Beveren C., Nagulapalli S., Klemsz M.J., McKercher S.R., Maki R.A. (1993). Effect of PU.1 phosphorylation on interaction with NF-EM5 and transcriptional activation. Science.

[45] Eisenbeis C.F., Singh H., Storb U. (1995). Pip, a novel IRF family member, is a lymphoid-specific, PU.1-dependent transcriptional activator. Genes Dev..

[46] Su G.H., Ip H.S., Cobb B.S., Lu M.M., Chen H.M., Simon M.C. (1996). The Ets protein Spi-B is expressed exclusively in B cells and T cells during development. J. Exp. Med..

[47] Escalante C.R., Brass A.L., Pongubala J.M., Shatova E., Shen L., Singh H. (2002). Crystal structure of PU.1/Irf-4/DNA Ternary Complex. Mol. Cell.

[48] Berndt S.I., Skibola C.F., Joseph V., Camp N.J., Nieters A., Wang Z. (2013). Genome-wide association study identifies multiple risk loci for chronic lymphocytic leukemia. Nat. Genet..

[49] Speedy H.E., Di Bernardo M.C., Sava G.P., Dyer M.J., Holroyd A., Wang Y. (2014). A genome-wide association study identifies multiple susceptibility loci for chronic lymphocytic leukemia. Nat. Genet..

[50] Landau D.A., Tausch E., Taylor-Weiner A.N., Stewart C., Reiter J.G., Bahlo J. (2015). Mutations driving CLL and their evolution in progression and relapse. Nature.

[51] Robbe P., Ridout K.E., Vavoulis D.V., Dreau H., Kinnersley B., Denny N. (2022). Whole-genome sequencing of chronic lymphocytic leukemia identifies subgroups with distinct biological and clinical features. Nat. Genet..

[52] Sundararaj S., Seneviratne S., Williams S.J., Enders A., Casarotto M.G. (2021). Structural determinants of the IRF4/DNA homodimeric complex. Nucleic Acids Res..

[53] Chang C.C., Lorek J., Sabath D.E., Li Y., Chitambar C.R., Logan B. (2002). Expression of MUM1/IRF4 correlates with clinical outcome in patients with B-cell chronic lymphocytic. Leuk. Blood.

[54] Ma S., Shukla V., Fang L., Gould K.A., Joshi S.S., Lu R. (2013). Accelerated development of chronic lymphocytic leukemia in New Zealand Black mice expressing a low level of interferon regulatory factor 4. J. Biol. Chem..

[55] Kim D., Paggi J.M., Park C., Bennett C., Salzberg S.L. (2019). Graph-based genome alignment and genotyping with HISAT2 and HISAT-genotype. Nat. Biotechnol..

[56] Xia Y., Chu W., Qi Q., Xun L. (2015). New insights into the QuikChange process guide the use of Phusion DNA polymerase for site-directed Mutagenesis. Nucleic Acids Res..

[57] Vizcaino J.A., Deutsch E.W., Wang R., Csordas A., Reisinger F., Rios D. (2014). ProteomeXchange provides globally coordinated proteomics data submission and dissemination. Nat. Biotechnol..

[58] Cox J., Neuhauser N., Michalski A., Scheltema R.A., Olsen J.V., Mann M. (2011). Andromeda: a peptide search engine integrated into the MaxQuant environment. J. Proteome Res..

[59] Tyanova S., Temu T., Sinitcyn P., Carlson A., Hein M.Y., Geiger T. (2016). The Perseus computational platform for comprehensive analysis of (prote)omics data. Nat. Methods.

[60] Dardis C. (2018).

[61] Therneau T.M. (2024).

