# Supplementary material for: Insights into the transcriptional regulation of CD22 in B cell chronic lymphocytic leukemia
Source: J Biol Chem. 2025 Mar 5;301(4):108386. doi: 10.1016/j.jbc.2025.108386 (PMC11999274; doi:10.1016/j.jbc.2025.108386)
Supplement: Supporting info.pdf [file mmc1.pdf]

Supporting information

Insights into the transcriptional regulation of *CD22* in B cell chronic lymphocytic leukemia

Bayarmaa Enkhbayar, Shao-Chia Lu, Ho-Yang Tsai, Suh-Yuen Liang, Shang-Ju Wu, Kuo-I Lin, and Takashi Angata

Table of contents Page

Supplementary methods	S-1
Figure S1	S-3
Figure S2	S-4
Figure S3	S-5
Figure S4	S-6
Figure S5	S-7
Figure S6	S-8
Figure S7	S-9
Table S1	S-10
Table S2	Separate Excel file
Table S3	S-11

Supplementary methods

Preparation of transcription factor constructs

The epitope-tagged transcription factor constructs were prepared as below. The primer sequences are provided in the Supplementary Table 1.

HA-tagged PU.1 expression construct: A full-length cDNA of PU.1 was cloned from the MEC-1 cell line by PCR, and an N-terminal HA tag was introduced by PCR with a forward primer containing the HA tag-coding sequence (F_HA_PU.1(NheI) + R_PU.1(EcoRI)). The PCR product was cloned into pLAS5w.Ppuro lentiviral transfer vector plasmid (RNA Technology Platform and Gene Manipulation Core, Academia Sinica).

HA-tagged IKZF3 expression construct: A full-length cDNA of IKZF3 was cloned from the MEC-1 cell line by PCR, and an N-terminal HA tag was introduced by PCR with a forward primer containing the HA tag-coding sequence (F_HA_IKZF3(NheI) + R_PmeI_IKZF3). The PCR product was cloned into pLAS5w.Ppuro plasmid.

HA-tagged IRF4 expression construct: A full-length cDNA of IRF4 was cloned from the JVM-3 cell line by PCR, and an N-terminal HA tag was introduced by PCR with a forward primer containing the HA tag-coding sequence (Forward_EcoRV_HA_IRF4 + Reverse_EcoRI_IRF4). The PCR product was cloned into pLAS5w.Ppuro plasmid.

Flag-tagged PAX5 expression construct: A full-length cDNA of PAX5 was cloned from the MEC-1 cell line by PCR (PAX5_F(NheI) + PAX5_R(EcoRI)) and cloned into pLAS5w.Ppuro plasmid. An N-terminal Flag tag was introduced by the PCR-amplification (F_flag_EcoRV + R_NheI_flag) of the Flag tag-coding segment of p3×FLAG-CMV-10 (Sigma) and cloning into the EcoRV-NheI sites of pLAS5w.PAX5.Ppuro plasmid.

Flag-tagged IKZF1 expression construct: A full-length cDNA of IKZF1 was cloned from the MEC-1 cell line by PCR. An N-terminal Flag tag was introduced by the PCR-amplification of IKZF1 ORF (HindIII_forward_IKZF1 + EcoRI_reverse_IKZF1) and cloning into p3×FLAG-CMV-10 plasmid (Sigma), then PCR-amplification of the cDNA including the Flag tag (Forward_NheI_flag_IKZF1 + Reverse_flag_IKZF1) and cloning into pLAS5w.Ppuro plasmid.

Flag-tagged IRF4 expression construct: Flag tag was added into IRF4 using NEBuilder HiFi DNA Assembly Cloning Kit with primer pairs "3xflag_forward" + "3xflag_reverse" and "forward_IRF4-2" + "reverse_IRF4-2".

Myc-tagged SPIB expression construct: A pcDNA3.1(-) containing an N-terminal Myc tag was prepared by cloning the annealed "XbaI_myc-tag_XhoI_forward" and "XbaI_myc-tag_XhoI_reverse" oligos into Xba I and XhoI sites of pcDNA3.1(-). A full-length cDNA of

SPIB was cloned from the MEC-1 cell line by PCR. N-terminally Myc-tag SPIB cDNA was prepared by cloning the full-length SPIB cDNA into the XhoI-EcoRI sites and further subcloning into pLAS5w.Ppuro plasmid.

Myc-tagged POU2F1 expression construct: A full-length cDNA of POU2F1 was cloned from the MEC-1 cell line by PCR. The PCR product was cloned into myc_pcDNA3.1(-) prepared above using XhoI and BamHI. A third PCR was performed (Forward_EcoRV_pcDNA + Reverse_NsiI_pcDNA) to introduce EcoRV and NsiI restriction sites, and the product was cloned into pLAS5w.Ppuro plasmid.

Myc-tagged RUNX3 expression construct: A Full-length cDNA of RUNX3 was cloned from the MEC-1 cell line by PCR. The PCR product was cloned into myc_pcDNA3.1(-) prepared above using XhoI and EcoRI, and the Myc-tagged RUNX3 cDNA was further subcloned into pLAS5w.Ppuro.

IRF4 point mutant: The Asp117 of IRF4 (involved in interaction with PU.1 and SPIB) was mutated to His using Q5 Site-Directed Mutagenesis Kit (E0554S, New England Biolabs). The PCR product was treated with KLD Mix (at room temperature, 10 minutes) and transformed into NEB Stable Competent E.coli (C3040H, New England Biolabs).

Recombinant lentivirus preparation

The 293T cells on 6 cm dishes (80-90% confluent) were transfected with the transfer vector construct (2.5 µg) along with packaging constructs (2.25 µg of pCMVΔR8.91 and 0.25 µg of pMD.G) and 15 µl Lipofectamine 2000 (11668019, Thermo Fisher Scientific). The medium was replaced with a fresh medium on the next day, and the supernatant was collected 24 and 48 hours after the replacement of the medium. The collected supernatant was aliquoted to 1 ml aliquotes and stored at −80°C until use.

Lentiviral transduction

Cells were seeded at the density of 0.5×10^6 cells/ml in a 24-well plate with polybrene (10 µg/ml; H9268, Sigma). Lentivirus (1 ml) was added to the cells, followed by centrifugation of the plate at 1000×g at 32°C for 30 minutes. The cells were incubated overnight in a humidified incubator at 37°C with 5% CO_2. On the next day, the culture medium was replaced by fresh medium, and a selective agent was added. The cells were selected for 1.5 to 2 weeks.

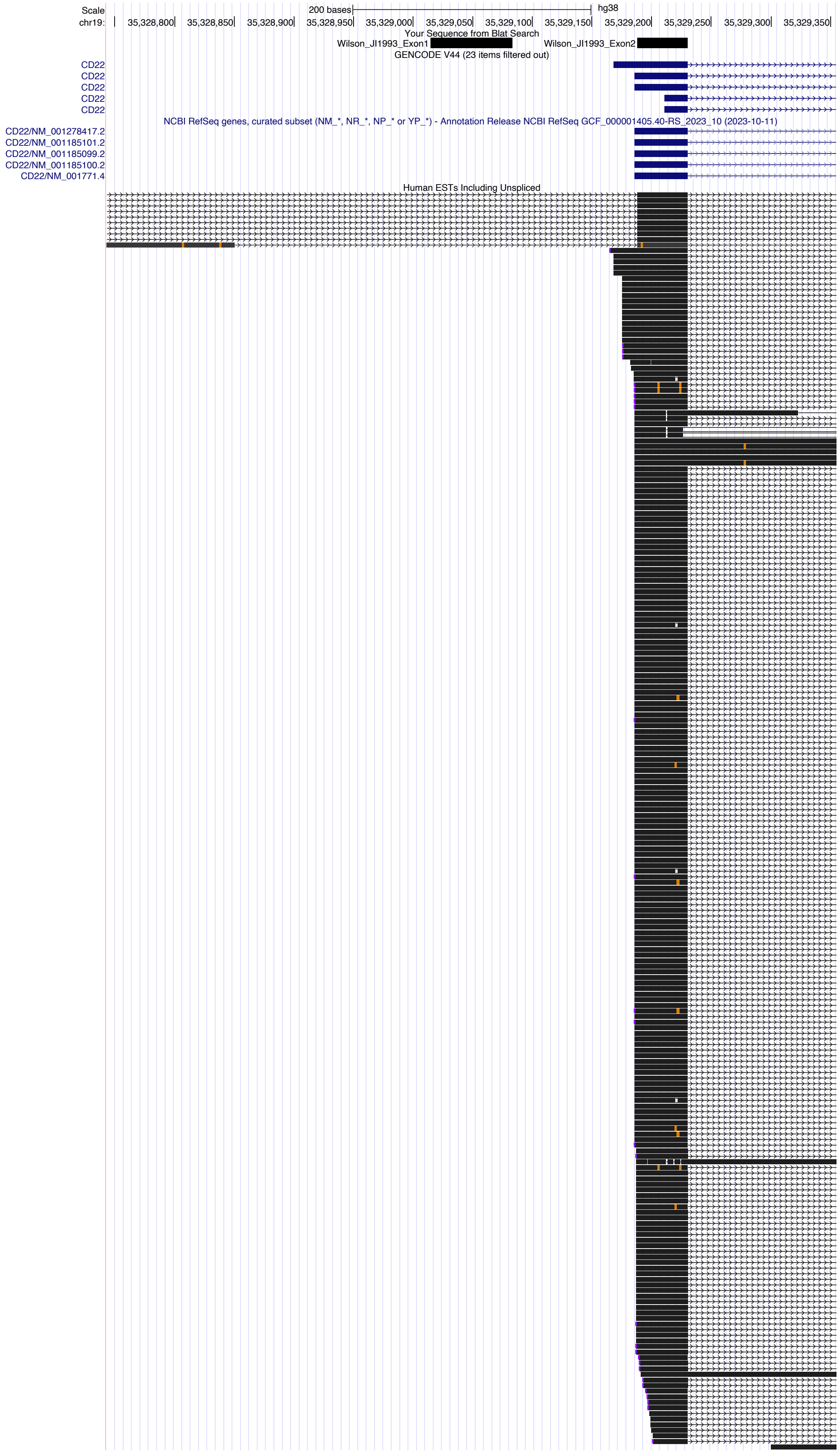

Figure S1. Alignment of the 5' ends of the *CD22* transcripts with the human genomic DNA sequence. The 5' end of the *CD22* transcript defined by Wilson et al. (25) was aligned by the BLAT algorithm to the human genome DNA sequence at the UCSC Genome Browser. Tracks for the reference transcripts (defined by GENCODE and NCBI) and expressed sequence tags (ESTs) were also included in the display. The exon 1 defined by Wilson *et al.* is not found among human *CD22* transcripts.

A

```
TTACTTTGTTGCCTGGGCTGGAGTGCAGTGGTGCTATCATGGCTGACTGAAGCCTTGAACTCCCAGGC
TCAAGCGATCCTCCTGCCTCAGCCTCCCAAGTAGTTTGGACCACAGGCATGGGCCACCATGCCCAGCT
ACTTTTCATTTTTTGCTTTTGTTGACTAGCCCCACAGAGTTTTGCTATGTTGTTCAGGTTGGTCTCAA
ACTTCTGGCCTTAAGCAATCCTCTTGCCTTGCTCTCCCAAAGCGTATTACAGGCGTAAGTCACCACAC
CCAGCCCTATCGGTGCTTTTGGAAGAAGGTGTTCTTCCCTCTCTCTGTTTCCCTCCCTATGGCCAGCT
GCCCATAGAGGGCAGTGGTGCTGGAGTGAAGACCTCTCAGCGCATCCCTCTTCATCTGTAGGATGTGC
ATGGCTTCCAATGCTGTCTGTTCTGCTCCTGAGACACTCGCTGGCTACAGGATTATAAAGGTGATGAG
GTTTCAGCCTAAAGACTCTCCATTCAATAACACTTCCTACAAGATATCTTTTTTGTTTTTTTGTTTTT
TTGAGACAGGATCTGGCTCTGTCACCCAGGCTGGAGTGCCACAGCGCGATCTGCTCACTGCAACCTCA
GCCTCTCGGGCTTAAGTGATTCTCCTGCCTTAGCCTCCTGAGTAGCTGGGACCACAGGCGCCCGCCAC
CACACCCAGCTAATTTTTGGATTTTTTTTTTTTTTTTTTAGTAGAGATGGGGTTTTACCATGTTGCCC
AAGCTGGTCTTGAACTCCTGAGCTCAGGCAATCCACCCGCCTTGGCCACCCAAAGCGCTAGGATTACA
GGCATGAGCCACCATGCCCAGTCCTACAAGATATCTTTTCTTTTTCTTCCTTTCTTTCTTTCTTTCTT
TTTTTTTTTTTGAGACAGAGTCTCACTCTGTCACCCAGGCTGGACTGCAGTGGTGTGATCTCTTCTCA
CTGCAGCCTCCGCCTCCTGGGCTCAAGTGATTCTCCTGCCTCAGCCCCCTGAGTAGCTGGGACTACAG
GCGCCTGCCAAAACACTTAGCTAATTTTTTGTATTTTTAGTAGAGACGGAATTTCACCATGTTCGCCA
GGCTGGTCTCGAATTCCTGACCTCAAGTGATCCACCCGCCTCAGCCTCCCAAAGTGTTGGGATTCCAG
GCATGAGCCACCGCACCCGGCCGAGATATCTTTTTCCTCCTCAATATCTATGTGCTCCTCCCTCAACC
CTTGGGGGGCTTCAGTGTAGGTGGTCAGATTCCCCACCATTTATGCCTATGATGAAAAGAGTGGAAAT
GGTAATAACTGGATGGTGTCACTAAACTACAAATACTTTCAGGTAGGCCTGTGCCTTTTTCAGCGGGC
TGCAGTTCTCCTGCTTGGCTTGAGTCATTCGCATTTCCTGAGAGCTGGGTAGAGGGGGAGTTGTGGAG
GAGCCCATTCTGAAACTGATCTGATATTGCAAACCCATACACGGAAAGGAAGAACTGCCCATACGTAT
TCGAGTCTTCCAATCTGACTCCGAGCCCGCATCCCCCTCAATCGTCTCTTTTCCCCACTCCCCAGATC
ACGGTGCTGCTTGGTCCCCCTCAGAGCCATAGAGAAGCAGGGGGTGTGGCCATGGAGGGGAAACCTCT
GTCACCAGAGACTTTACTGTACTTCTCCTTTTGCTCTCAGATGCTGCCAGGGTCCCTGAAGAGGGAAG
ACACGCGGAAACAGGTAAAAATCATTTTGCTTTTATTTTGCATTCAACAAGCAAGTTATTACGGAACA
GCAGTTATGGGCCAGGCATACCTCCCAGAGCTGGGAACACAGTGGGGACCTCCCTGGCTCTCTCTTAC
CGGTGTTACAACAGGTTGTAGACAGACCCCTGTCTTGAGCATCCTCCTTGCCAGGCCTGCTGAGTCTT
CTGAGAGTAG
```

A: TSS by Wilson et al.
Underline: +88/+176
Blue shade: GC-box
Yellow shade: E-box

B

```
WT      AGAGCCATAGAGAAGCAGGGGGTGTGGCCATGGAGGGGAAACCTCTGTCACCAGAGACTT
GC_mut  AGAGCCATAGAGAAGCAGGtacTcTGGCCATGGAGGGGAAACCTCTGTCACCAGAGACTT
E_mut   AGAGCCATAGAGAAGCAGGGGGTGTGGCCATGGAGGGGAAACCTCTGTCACCAGAGACTT

WT      TACTGTACTTCTCCTTTTGCTCTCAGATGCT
GC_mut  TACTGTACTTCTCCTTTTGCTCTCAGATGCT
E_mut   TACTGTACTTCTCCTTTTGCTCTacGcTtCT
```

Figure S2. Sequences of the promoter construct and the probes.
(A) Sequence of the longest *CD22* promoter (-1,499/+415) used in the study. (B) Sequences of the DNA probes used for DNA pulldown-proteomics. The minimal *CD22* promoter (+88/+178) and GC-box/E-box mutants are aligned. Substituted nucleotides are highlighted in green.

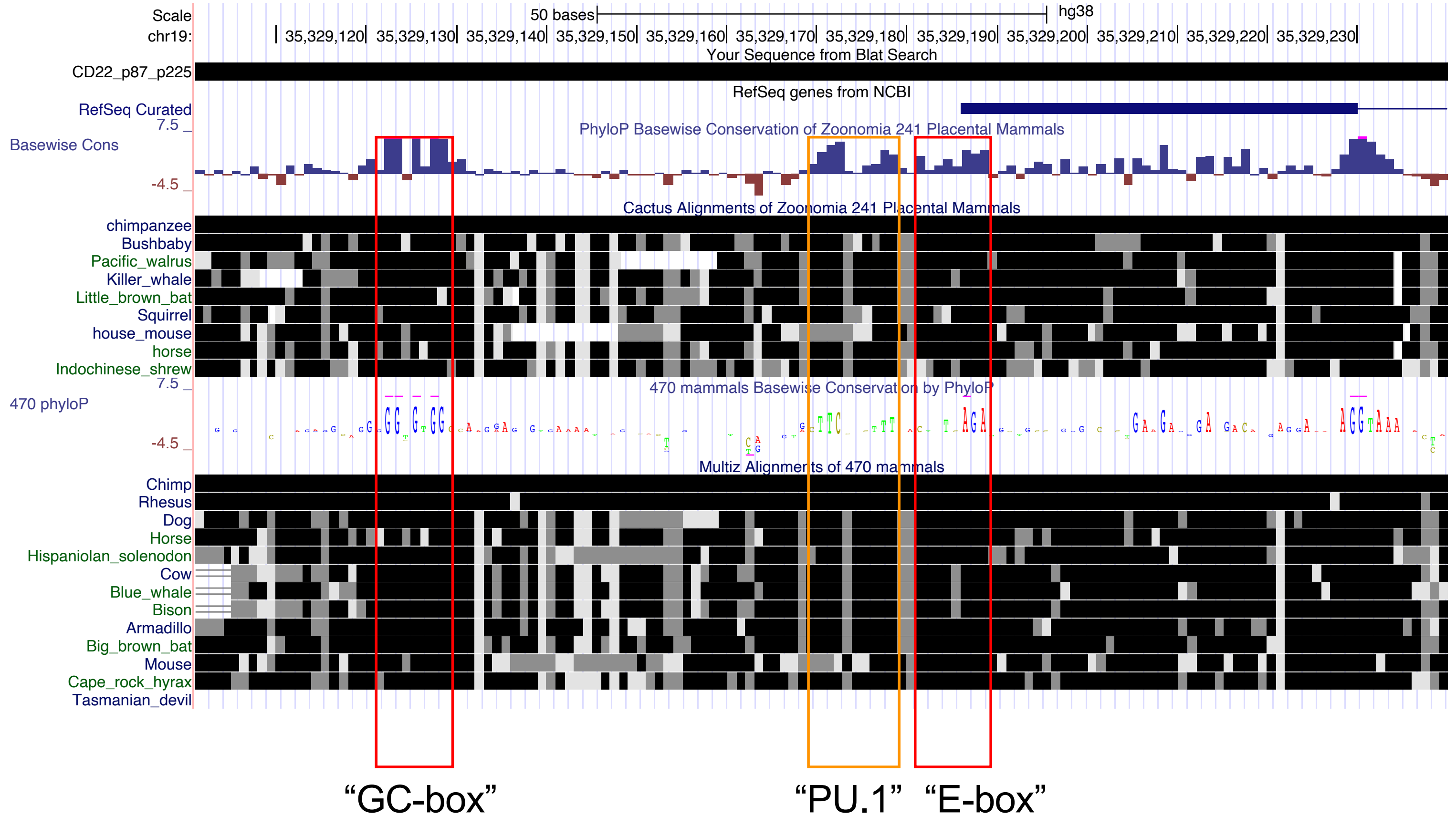

Figure S3. Conservation of genomic DNA sequences near the CD22 transcriptional start site. Tracks for “Cactus Alignment & Conservation of Zoonomia Placental Mammals (241 Species)” (29) and “Multiz Alignment & Conservation (470 mammals)” near the CD22 transcriptional start site are displayed in the UCSC Genome Browser. Conserved sequence elements, i.e., putative “GC-box”, “PU.1 binding site” and “E-box” motifs are indicated.

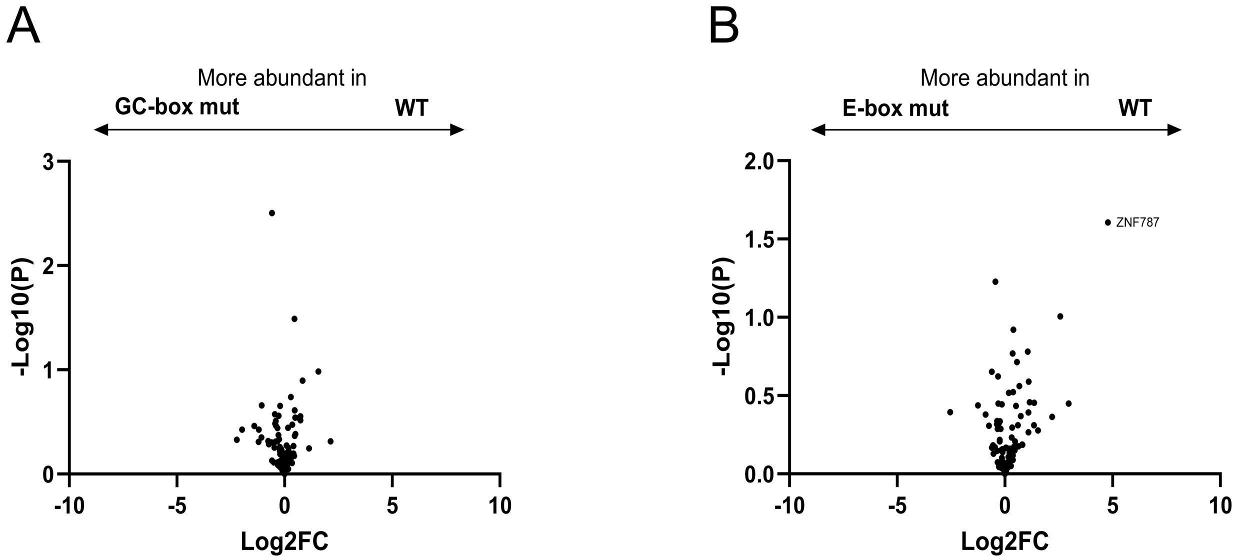

Figure S4. DNA pulldown-proteomics with MEC-1 nuclear extracts.
Comparisons of proteins (A) extracted from MEC-1 with wild-type *versus* GC-box mutant probes and (B) extracted from MEC-1 with wild-type *versus* E-box mutant probes are shown. Only a few differences in the transcription factors bound to mutant probes were found.

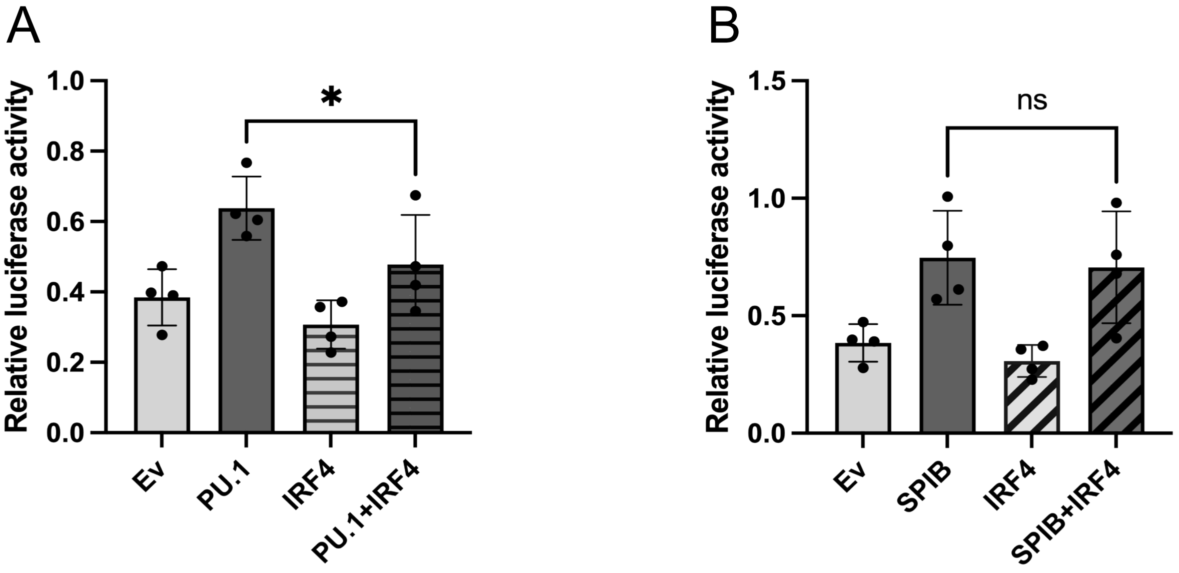

Figure S5. Luciferase reporter assay using PU.1, SPI-B, and IRF4.
The 293T cells were transiently transfected with the reporter constructs (pGL4.12[luc2CP] with minimal *CD22* promoter and pGL4.74[hRluc/TK]) with the expression constructs for PU.1, Spi-B, and/or IRF4, and subjected to luciferase reporter assays. The firefly luciferase activity was normalized with that of the *Renilla* luciferase in the same sample. IRF4 suppressed reporter transactivation by PU.1, whereas it did not have a strong effect on that by Spi-B. $^*P < 0.05$ (paired t-test). $n = 4$ (technical replicates).

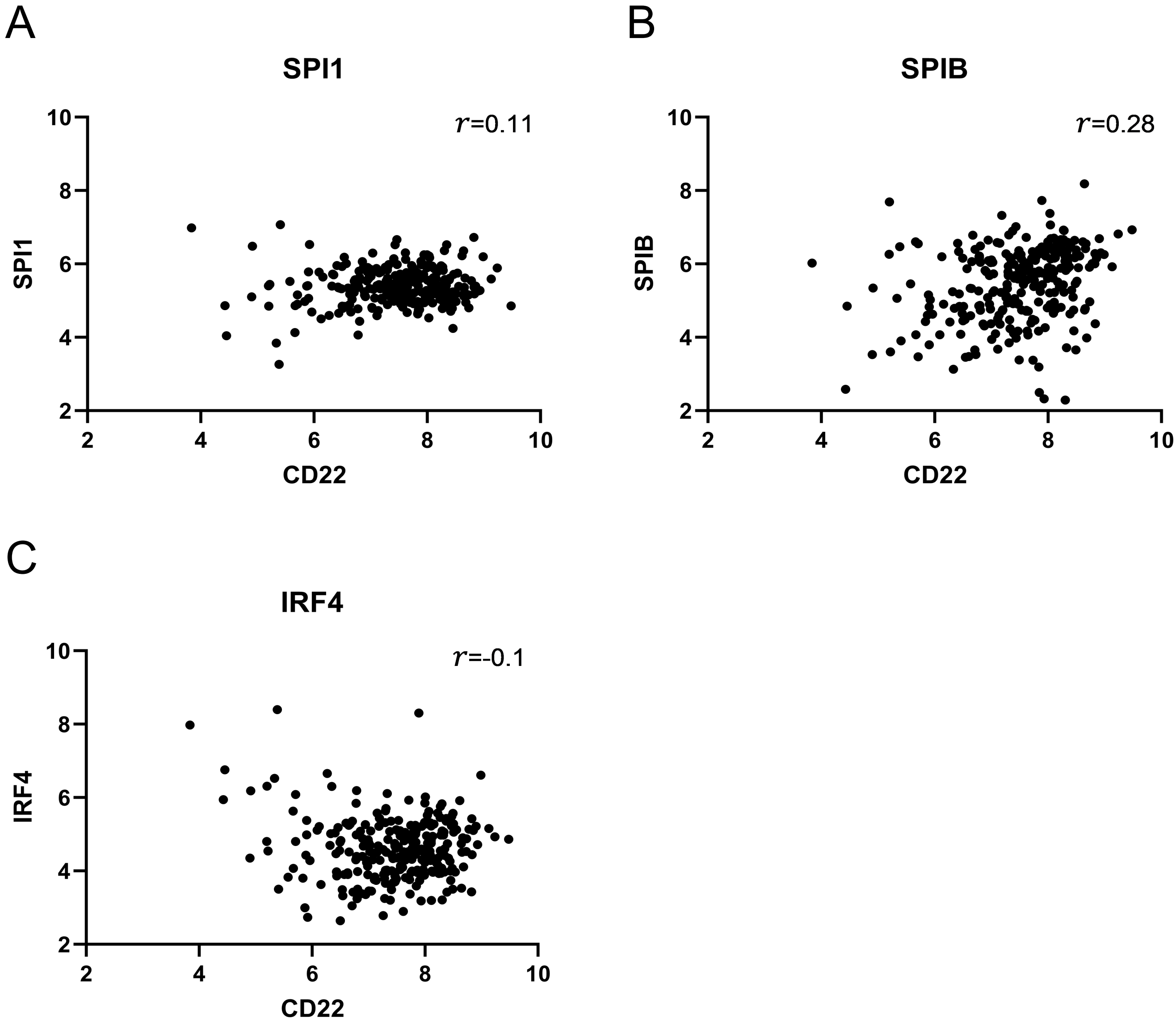

Figure S6. Associations between the transcript levels of *CD22* and transcription factors.
Transcript levels for *CD22* and (A) *SPI1* (encoding PU.1), (B) *SPIB*, and (C) *IRF4* were extracted from the RNAseq dataset for Spanish CLL patients (*n* = 263) (27,28) and plotted. Pearson's correlations between the log2-transformed transcript levels for *CD22* and the transcription factors were calculated. *CD22* transcript level was positively correlated with those of *SPI1* and *SPIB* but was negatively correlated with that of *IRF4.*

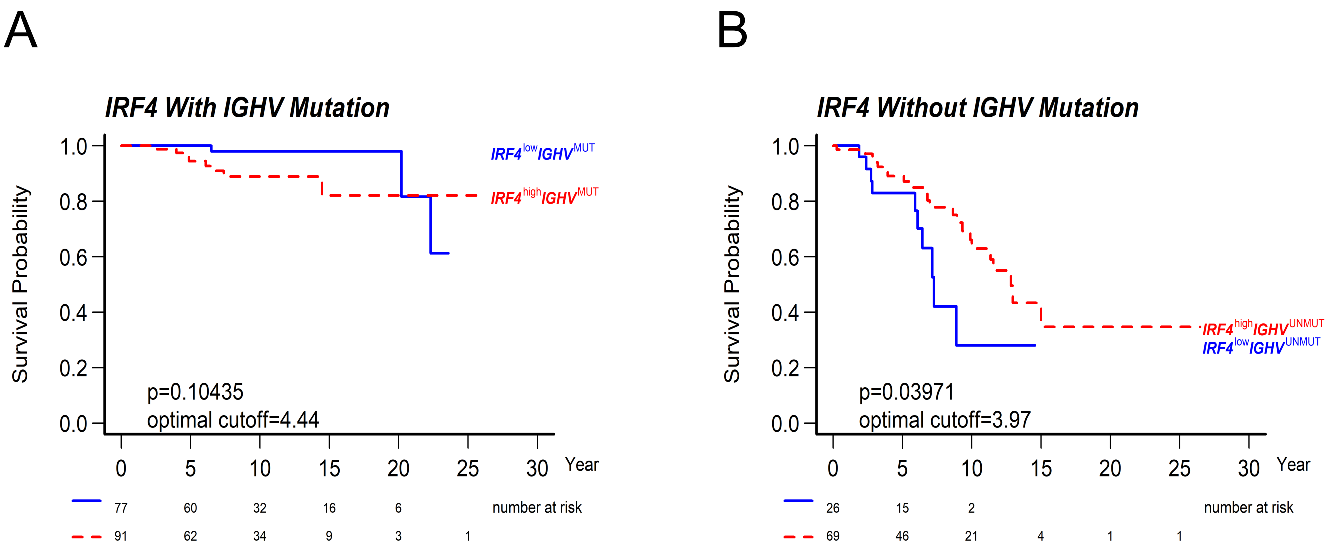

Figure S7. The *IRF4* transcript level and CLL patient survival.
The transcriptomic dataset of Spanish patients with CLL was stratified into those with or without mutations in *IGHV*, and the correlation between the *IRF4* transcript level and patient survival was plotted for each group. (A) Patients with *IGHV* mutations (n = 168). (B) Patients without *IGHV* mutations (n = 95). The optimal cut-off was determined for each group with the cutp function in survMisc (60), an R package.

Table S1. Primers used

Name	Sequence (5' to 3')
Cloning primers	
CD22 promoter -1499 KpnI F	AAGGTACCTTACTTTGTTGCCTGGGCTG
CD22 promoter -362 KpnI F	AAGGTACCCAAAGTGTTGGGATTCC
CD22 promoter -163 KpnI F	AAGGTACCAGGCCTGTGCCTTTTTCAG
CD22 promoter -50 KpnI F	AAGGTACCTGATATTGCAAACCCATACAC
CD22 promoter +28 KpnI F	AAGGTACCATCCCCCTCAATCGTCTC
CD22 promoter +88 KpnI F	AAGGTACCAGAGCCATAGAGAAGCAGG
CD22 promoter +117 KpnI F	AAGGTACCATGGAGGGGAAACCTCTG
CD22 promoter +415 XhoI R	TCTACTCGAGCTACTCTCAGAAGACTCAGCAGG
CD22 promoter +176 XhoI R	TCTACTCGAGCATCTGAGAGCAAAAGGAG
CD22 promoter +134 XhoI R	TCTACTCGAGCAGAGGTTTCCCCTCCATGG
CD22 promoter +71 XhoI R	TCTACTCGAGCACCGTGATCTGGGGAGTG
Forward_SPIB	AAACAGCCCGCCCGG
Reverse_SPIB	ACGTCCCAGCCAGGATC
XhoI_forward_SPIB	AAACTCGAGATGCTCGCCCTGGAGGCTG
EcoRI_reverse_SPIB	GTTGAATTCCTCAGGCCCGGCGGACT
XbaI_myc-tag_XhoI_forward	CTAGAGCCACCATGGAACAAAAACTCATCTCAGAAGAGGATCTGC
XbaI_myc-tag_XhoI_reverse	TCGAGCAGATCCTCTTCTGAGATGAGTTTTTGTTCCATGGTGGCT
F_HA_PU.1(NheI)	AAAGCTAGCGCCACCATGTACCCATACGATGTTCCAGATTACGCTATGTTACAGGCGTGCAAAATGG
R_PU.1(EcoRI)	AAAGAATTCTTAATGCTATGGCCAGCG
PAX5_F(NheI)	AAAGCTAGCGTCCATTCCATCAAGTCCTG
PAX5_R(EcoRI)	CCCCGAATTCTCAGTGACGGTCATAGGCAG
F_flag_EcoRV	AAAGATATCGCCACCATGGACTACAAAGACCATGACG
R_NheI_flag	AAAGCTAGCCTTGTCATCGTCATCCTTG
Forward_IKZF1	CGCACAAATCCACATA
Reverse_IKZF1	CCAGGAAAAGCACAAGG
HindIII_forward_IKZF1	AACAAGCTTGCCACCATGGATGCTGATGAGGG
EcoRI_reverse_IKZF1	GTTGAATTCCTTTAGCTCATGTGGAAGCG
Forward_NheI_flag_IKZF1	AACGCTAGCCACCATGGACTACAAAGACCA
Reverse_flag_IKZF1	GCAACTTCCAGGGCCAGG
IKZF3_forward	GACTAGCCACAGCGGCAGCTC
IKZF3_reverse	CCTGGCTACCCCTGTGAACACAGC
F_NheI_IKZF3	AAAGCTAGCGCCACCATGGAAGATATACAAACAAATGCGG
R_PmeI_IKZF3	AAAGTTTAAACTCACTTCAGCAGGGCTCTGT
F_HA_IKZF3(NheI)	AAAGCTAGCGCCACCATGTACCCATACGATGTTCCAGATTACGCTATGGAAGATATACAAACAAATGCGG
FP_POU2F1	GCTTAAGAACATACTGTAGATTTG
RP_POU2F1	GTTCTCCAATCCATGAAGC
XhoI_POU2F1_FP	AAACTCGAGATGCTGGACTGCAGTGACTAT
POU2F1_BamHI_RP	AAAGGATCCTCACTGTGCCTTGGAGG
Forward_EcoRV_pcDNA	AAAGATATCCCACTGCTTACTGGCTTATCG
Reverse_NsiI_pcDNA	AAAATGCATGACACCTACTCAGACAATGCG
F_RUNX3_tv2	CTTCTGCTTTCCCGCTTCTCG
R_RUNX3_tv2	CTGGAGCGCAGGTCCCATT
F_RUNX3_tv2 (XhoI)	AAACTCGAGATGCGTATTCCCGTAGACCCAA
R_RUNX3_tv2 (EcoRI)	AAAGAATTCTCAGTAGGGCCGCCACAC
Forward_IRF4	GTTTCACCGCTCGATCTTGG
Reverse_IRF4	GATTGTGTCACTGCACTCCAGC
Forward_EcoRV_HA_IRF4	GATATCGCCACCATGTACCCATACGATGTTCCAGATTACGCTATGAACCTGGAGGGC
Reverse_EcoRI_IRF4	GAATTCTCATTCTTGAATAGAGG
3xflag_forward	TAGGGATATCCACGTGGCGATCGCCACCATGGACTACAAAGAC
3xflag_reverse	TCCAGGTTCATGTCATCGTCATCCTTGTAATC
Forward_IRF4-2	ATGACGATGACATGAACCTGGAGGGCGGCGG
Reverse_IRF4-2	CATACGCGTCCTGCAGGCGATCTCATTCTTGAATAGAGGAATGGCGGATAGATC
Mutagenesis primers	
Forward_pCD22_mutEbox	TTGCTCTACGCTTCTCGAGGATATCAAGATC
Reverse_pCD22_mutEbox	CCTCGAGAAGCGTAGAGCAAAAGGAGAAG
Forward_pCD22_mutGCbox	AGCAGGGAATATTGCCATGGAGGGGAAACC
Reverse_pCD22_mutGCbox	CCATGGCAATATTCCCTGCTTCTCTATGGCTC
Forward_pCD22_mSPIB,mPU.1	GCACTATCCCTCAGATGCTCGAGGATATC
Reverse_pCD22_mSPIB,mPU.1	ATCATTAAAGTAAAGTCTCTGGTGACAG
Forward_IRF4D117H	GAGCCAGCTGCATATCTCAGACC
Reverse_IRF4D117H	CGCTCAACCAGTTCC
Preparation of biotinylated DNA probe	
Forward_CD22_probe	Biotin-AAAAAATGAATTCTGGGGGCCTCAGTATC
Reverse_CD22_probe	ATGTCTCCATCCGCCAGG
Quantitative PCR	
Forward_pCD22	AGAGCCATAGAGAAGCAGGG
Reverse_pCD22	AGCATCTGAGAGCAAAAGGAGAAG
qPCR_probe_pCD22	FAM-CATGGAGGGGAAACCTCTGTCACC-NFQ

Red: restriction enzyme recognition site; Blue: epitope tag coding sequence; Green: chemical modification

Table S3. Antibodies used

Antigen	Vendor	Catalog number	Clone	Applications
CD22	Biolegend	302510	HIB22	Flow cytometry
POU2F1	Cell Signaling Technology	8157	D7B6	ChIP, WB
RELB	Cell Signaling Technology	10544	D7D7W	ChIP
SPI1	Cell Signaling Technology	2266	(Polyclonal)	ChIP, WB
SPIB	Cell Signaling Technology	14337	D4V9S	ChIP, WB
IKZF1	Cell Signaling Technology	14859	D6N9Y	ChIP, WB
IKZF3	Cell Signaling Technology	15103	D1C1E	ChIP, WB
IRF4	Cell Signaling Technology	4964	(Polyclonal)	ChIP, WB
PAX5	Invitrogen	14-9918-82	1H9	ChIP, WB
RUNX3	Biolegend	653604	9F4A17	ChIP, WB
STAT3	Cell Signaling Technology	9139	124H6	ChIP
pSTAT3 (Tyr705)	Cell Signaling Technology	9131	(Polyclonal)	ChIP
HA-tag	Abcam	ab130275	16B12	EMSA, WB
Myc-tag	Invitrogen	MA1-21316	Myc.A7	EMSA, WB
FLAG-tag	Sigma	F3165-5MG	M2	EMSA, WB
β-actin	Invitrogen	MA5-15739-HRP	BA3R	WB
